# Unsupervised Learning of Cone Spectral Classes from Natural Images

**DOI:** 10.1371/journal.pcbi.1003652

**Published:** 2014-06-26

**Authors:** Noah C. Benson, Jeremy R. Manning, David H. Brainard

**Affiliations:** 1 Department of Psychology, University of Pennsylvania, Philadelphia, Pennsylvania, United States of America; 2 Department of Neurology, University of Pennsylvania, Philadelphia, Pennsylvania, United States of America; University of Tübingen and Max Planck Institute for Biologial Cybernetics, Germany

## Abstract

The first step in the evolution of primate trichromatic color vision was the expression of a third cone class not present in ancestral mammals. This observation motivates a fundamental question about the evolution of any sensory system: how is it possible to detect and exploit the presence of a novel sensory class? We explore this question in the context of primate color vision. We present an unsupervised learning algorithm capable of both detecting the number of spectral cone classes in a retinal mosaic and learning the class of each cone using the inter-cone correlations obtained in response to natural image input. The algorithm's ability to classify cones is in broad agreement with experimental evidence about functional color vision for a wide range of mosaic parameters, including those characterizing dichromacy, typical trichromacy, anomalous trichromacy, and possible tetrachromacy.

## Author Summary

The human visual system encodes color by comparing the responses of three different kinds of photoreceptors: the long- (reddish), medium- (greenish), and short- (bluish) wavelength-sensitive cone cells. In order for the visual system to accurately represent the color of stimuli, it must (in effect) know the class of the cone that produced each response. The long- and medium-wavelength-sensitive cones, however, are virtually identical in every known way except that their responses to a given spectrum of light differ. Here, we simulate cones in a model human retina and show that by examining the correlation of the responses of cones to natural scenes, it is possible to determine both the number cone classes present in a retinal mosaic and to explicitly determine the class of each cone. These findings shed light on the computational mechanisms that may have enabled the evolution of human color vision, as well as on the more general question of whether and when it is possible for sensory systems to self-organize.

## Introduction

Primate color vision is initiated when light spectra entering the eye are encoded by three classes of retinal cone photoreceptors: the long- (L), medium- (M), and short- (S) wavelength-sensitive cones. Trichromatic encoding alone, however, is not sufficient to support functional trichromatic color vision. In addition, the information encoded by the cones must be preserved by post-receptoral neural circuitry in a manner that enables both color discrimination and the representation of stimulus color.

The emergence of distinct L and M cones in primates is a recent evolutionary event [1]–[4], and the only known difference between these cones is the photopigment opsin contained in their outer segments [5], [6]. These observations make it unlikely, given current knowledge, that the formation of appropriate post-receptoral circuitry can rely on biochemical markers that distinguish L and M cones and suggest instead that this circuitry must be learned.

The fact that the first step in the evolution of trichromatic color vision was the expression of a third cone class leads to two important questions. First, how can a post-receptoral visual system detect the presence of a new cone class? Second, how can such a system classify individual cones according to their spectral class, so as to take advantage of the information carried by the newly expressed opsin? More generally, the question of what information enables the brain to initially detect and differentiate the presence of novel sensory transducers applies to any sensory modality which provides a multidimensional perceptual representation. Here we provide a computational treatment of these fundamental questions in the context of primate color vision and show that the inter-cone correlations induced by natural images may be used to detect when a mosaic contains three rather than two cone classes and to accurately classify L versus M cones.

We build on previous work, some available only in abstract form, that considers the concrete question of how learning might produce model neural units whose wiring differentiates signals from L and M cones [7], [8]. Importantly, this work shows that there is indeed information in the cone responses to natural images that can differentiate L and M cones to some degree, in the sense that learning enabled above-chance formation of cone-class specific wiring of model units. Here, we extend the previous work in two ways.

First, we develop an algorithm that uses inter-cone response correlations to explicitly classify cones by their spectral type. Although such explicit classification is not a necessary feature of the brain's implementation of visual processing, it allows us to quantify the degree to which unsupervised learning can differentiate cones by type to support color vision. This quantification allows us to explore how classification performance depends on key parameters of the mosaic, for example particular on the number of cone types, the ratio of L to M cones, and the spectral sensitivities of the different cone classes.

Second, the appropriate representation of stimulus color depends on the number of cone classes present in the mosaic. In humans [9] (and also New World primates [2]) there is individual variation in this number, primarily in the form of red-green dichromacy. Thus it is also of interest to investigate whether learning can detect the number of longer-wavelength-sensitive cone classes present in an individual retinal mosaic.

Our approach is computational. That is, we abstract, for the most part, from known features of retinal and cortical circuitry and consider what is possible using information encoded by the distal retina in response to natural images.

## Results

**Figure 1:**
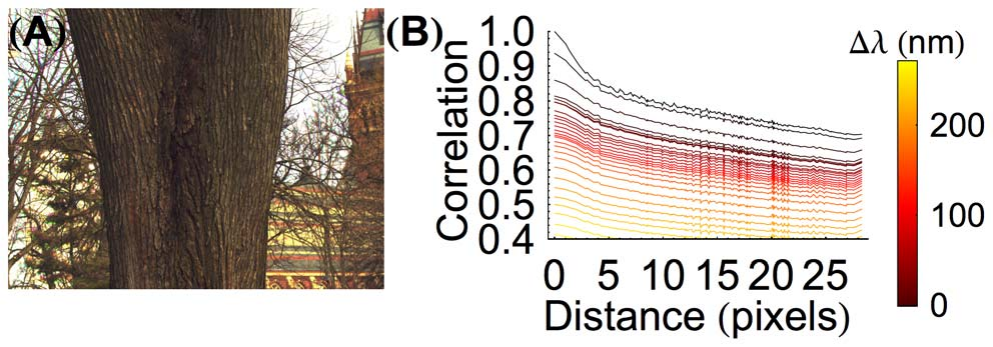
Natural image correlations are highly regular in both space and spectrum. (**A**) An RGB rendering of a hyperspectral image taken from the natural image database described by Chakrabarti and Zickler [12]. The code used to render this figure is included in our gitHub repository (https://github.com/DavidBrainard/ReceptorLearning/). (**B**) Image correlations for images from our combined database. Correlation is plotted as a function of distance (pixel). Each curve represents a different wavelength separation: the black curve represents no wavelength difference while the yellow curve represents a difference of 320 nm (*i.e.*, the 400 nm channel correlated with the 720 nm channel).

We begin by considering how to classify L versus M cones in a mosaic that is known to contain both cone classes; below we return to the problem of detecting the number of cone classes present in a mosaic. To proceed, we simulated the responses of retinal cone mosaics to natural images. The images used in the simulations were collected from three hyperspectral image databases [10]–[12]. A hyperspectral image specifies the full light spectrum at each pixel, in contrast to standard color images which contain only red, green, and blue (RGB) values. The use of hyperspectral images allows us to accurately simulate the responses of cones from their specified spectral sensitivities. An RGB rendering of a hyperspectral image from the dataset is shown in Figure 1A.

**Figure 2:**
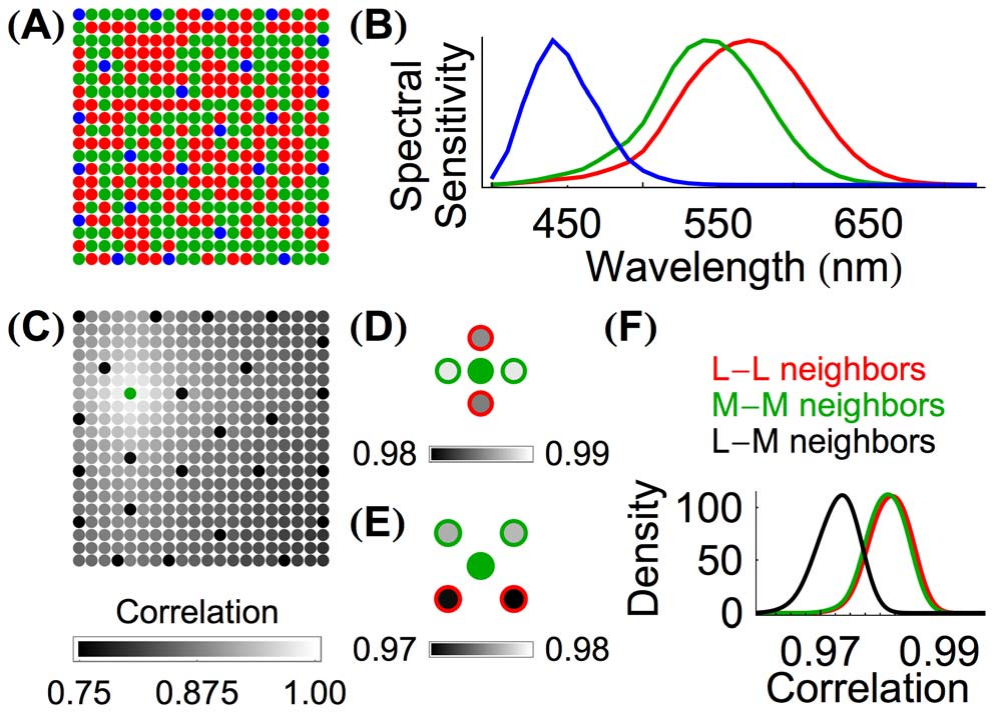
Inter-cone correlations carry information about cone spectral class and mosaic arrangement. (**A**) A 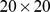 cone mosaic with an L∶M∶S ratio of 47∶47∶6; each cone is colored by class (red, green, and blue for L, M, and S cones, respectively). (**B**) The spectral sensitivities of L, M, and S cones in a typical fovea [52]. (**C**) The mosaic from Panel **A** with grayscale indicating the correlation of each cone's response to the response of the single M cone plotted in green. The correlations are calculated from the responses to 2,000,000 natural images. At this scale it is readily apparent that correlations decrease with inter-cone distance and that S cones are distinguished from M cones by their correlations. (**D**) Magnified view of the correlation of the immediately adjacent neighbors of the green M cone from Panel **C**, with grayscale indicating correlation. Red and green circles indicate the class of each neighboring cone. Of the four immediate neighbors of the central cone, the two neighboring M cones (green) have higher correlations than the neighboring L cones (red). (**E**) Magnified view of the correlation of the diagonal neighbors of the green M cone from Panel **C**, with grayscale indicating correlation. The neighboring M cones have higher correlations than the two neighboring L cones. (**F**) A smoothed histogram of the correlations of immediately neighboring longer-wavelength-sensitive cones in 54 simulated mosaics whose M cone 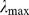 value was 530 nm and whose L cone 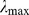 value was 558.9 nm. The 54 mosaics differed in their L∶M cone ratios and sizes. Correlations between neighboring L cones, neighboring M cones, and L-M neighbors are shown in red, green, and black respectively. The histograms represent correlations from 9,862 L-L cone pairs, 9,769 M-M cone pairs, and 8,369 L-M cone pairs.

We constructed simulated cone mosaics using parameters typical of human vision. Figure 2A illustrates one of our simulated mosaics. The assumed spectral sensitivities are shown in Figure 2B. We simulated the responses of each cone in the mosaic to 2 million image patches sampled randomly from our full hyperspectral image dataset and computed the 400 by 400 correlation matrix between the responses of all pairs of cones in the mosaic. Because the distal retina forms spatially antagonistic receptive fields [13], [14], our main analysis used simulated cone responses that incorporated an opponent surround that draws on neighboring cones irrespective of their type (see Methods).

Figure 2C depicts one row of the full correlation matrix in image format, with the gray level of each cone in the panel indicating the correlation between that cone and the single M cone shown in green. Two previously observed features of the correlation structure may be seen in this representation [7], [15], [16]. First, correlation in cone responses decreases as a function of spatial distance. Second, once distance is equated, correlations between cones of the same class are higher than correlations between cones of different classes (Figure 2D and 2E). These two features are induced by the spatial-spectral correlation structure of natural hyperspectral images (Figure 1B).

Figures 2D and 2E provide intuition about what information could be used to classify L versus M cones. Consider an illustrative algorithm that starts with a single cone (*e.g.*, the M cone shown in Figure 2C) and labels it (arbitrarily) as an M cone. The algorithm would then classify that cone's neighbors as being of the same or different class by setting a threshold on the correlation values. By propagating this procedure across the mosaic, labels would be assigned to all cones. Such an algorithm would be effective if the distributions of nearest neighbor within-class correlations had little if any overlap with the distribution of between-class correlations.

Figure 2F shows the histograms of such correlations for our dataset. Although the histograms make clear that within-class correlations are on average higher than between-class correlations and therefore carry information about cone class, the overlap between the two types of correlation means that nearest neighbor correlations alone are not sufficient for good classification. Note, however, that the histograms shown in Figure 2F only depict the information carried by nearest neighbor correlations, leaving open the possibility that additional information carried by the full correlation matrix could support a robust solution to the cone classification problem.

To visualize and exploit the information about cone class carried by the full correlation matrix, consider a three-dimensional representational space where the first two dimensions, call them 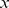 and 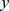, provide the spatial position of a cone in the mosaic and where the third dimension, call it 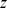, indicates spectral class. To conceive of 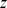 as continuous, we treat it as indicating the wavelength of peak sensitivity, 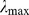, of each cone. Because the correlation between two cones decreases both with increasing spatial distance and with increasing 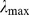 difference between them, the negative log of the correlation matrix may be thought of as a proxy for the distance between cones in this abstract representational space.

**Figure 3:**
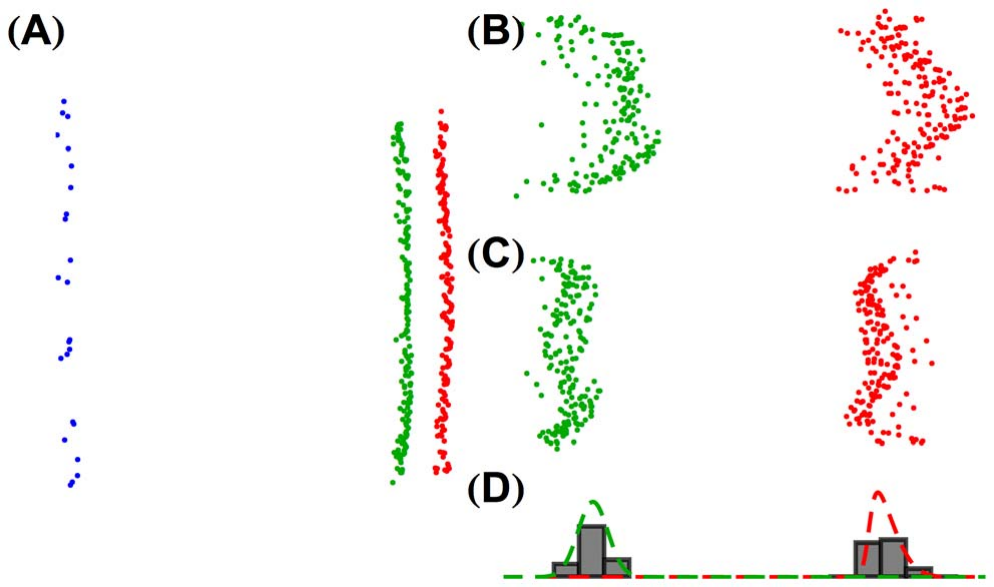
Multidimensional scaling allows classification of cones for a typical trichromatic retinal mosaic. (**A**) 3D embeddings of the correlation matrix of the 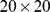 mosaic from Figure 2A. Each point represents a single cone and is colored red, green, or blue for L, M, or S respectively, according to its actual identity in the mosaic. The 3D embeddings shown here and in other figures in this paper are oriented so that the 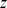-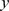 plane (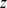 horizontal, 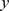 vertical) of the representational space described in the text is shown. The absolute units on these axes are not meaningful, because MDS solutions are determined only up to a relative-distance preserving transformation. (**B**) The same 3D embedding shown in A zoomed in on the embedding of the L and M cones only. (**C**) The 3D embedding of the L and M cones from **A** after flattening. (**D**) A histogram of the 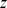 positions of the embedding from **C** (*i.e.*, after flattening); best fit skew normals are shown in red and green. Rotating animations that show the three-dimensional structure of the embeddings are available online (http://color.psych.upenn.edu/supplements/receptorlearning).

We used the well-developed technique of non-metric multidimensional scaling (MDS) [17] to place each cone from the mosaic shown in Figure 2A at a position in a three-dimensional space such that the inter-cone distances in this space best predicted, up to a free monotonic transformation, the negative log of the correlation matrix. Figure 3A shows the result. Each point represents a single cone, and the cones are colored red, green, or blue for L, M, and S respectively. It is clear that when the full correlation matrix is used to embed the mosaic cones, their positions separate strongly by class. Figure 3B shows an expanded view of the embedding that includes only the L and M cones. In both Figure 3A and 3B, the view is of the 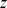-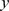 plane of the representational space. A view of the 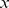-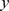 plane for the L and M cones is provided in Figure S1 and shows that the relative spatial positions of these cones are also well-preserved in the embedding. Figure S2 shows that the embedding accounts well for the underlying correlation matrix. Figure S3 shows embeddings for additional simulations where the separation of L and M cone spectral sensitivities and L∶M cone ratio were varied. These additional simulations are discussed in more detail below.

Note that MDS embeddings are determined only up to a distance preserving transformation. In our simulations, for 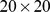 mosaics that contained S as well as longer-wavelength-sensitive cones, the first dimension of the embedding returned by the MDS algorithm we used (Matlab's mdscale, found in the Statistics Toolbox) generally corresponded approximately to the 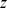 dimension of the conceptual representational space described above. This first dimension is by the conventions of MDS the one that explains most of the variance in the similarity matrix, and it is the large separation between the responses of S cones compared to longer-wavelength-sensitive cones that drives this alignment. Throughout this paper, then, we refer to the first, second, and third dimensions of the MDS solution as the 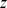, 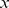, and 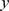 dimensions respectively. The only exceptions to this ordering of the dimensions were simulations of tritanopic (no S cones) mosaics and small (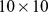 and 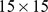) mosaics. When we simulated tritanopic mosaics and the smaller mosaics, the MDS solutions reliably separated cone type along the third instead of the first dimension, instead organizing the spatial arrangement of the mosaic along the first two dimensions. For all simulations of mosaics containing S cones, we used an algorithmic procedure to rotate the embedding returned by the MDS algorithm so that the first dimension of the rotated solution corresponded more precisely to the 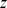 dimension of the conceptual representational space. The rotation algorithm is described in Methods. It relied on the fact that the S cones could be easily identified from the embeddings (again, see Methods) and it aligned the vector from the mean of the embedded positions of the non-S cones to the mean of the embedded position of the S cones. For all simulations with 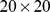 mosaics the effect of this rotation was small. We return to the analysis of learning for tritanopes below.

The embedding shown in Figure 3 separates the three cone classes along the 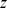 (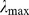) dimension of the representational space. The positions of the S cones are highly distinct from those of the L and M cones. The L and M cones also separate well from each other, although in other simulation conditions there is overlap in the 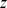 positions of these two cone classes (see, for example, Figure S3). In most such cases, however, the L and M cones are actually embedded along separate curved surfaces within the full-three dimensional representation, but the curvature of these surfaces produces overlap in the 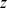 positions of L and M cones. The curved surfaces may be flattened in a manner that preserves the local planar structure of each (see Methods). The result of applying the flattening procedure to the embedding shown in Figure 3B is provided in Figure 3C, where the separation between the L and M clusters remains apparent. To classify cones, we extracted the 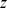 position of each cone in the flattened representation and fit the resulting distribution as the mixture of two skew normal distributions (Figure 3D). We then used the parameters of the two distributions to determine a threshold along the flattened 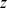-axis, and labeled all cones to the left of the threshold as M and all cones to the right as L. The threshold was taken as the point between the means of the two fit distributions where their probability density functions were equal. Note that the labeling of L versus M is not arbitrary, but rather is determined by the position of the well-separated S cones, which are themselves classified algorithmically through a preprocessing step (again see Methods). This algorithm classifies 100% of the L and M cones correctly from the correlation matrix, for the mosaic shown in Figure 2A.

Across individuals, human retinal mosaics vary in two key ways. First, there is substantial variation in the L∶M cone ratio, from approximately 1∶3 to 16∶1 [18]–[20]. Second, polymorphisms in the genes that encode the L and M cone opsins lead to variation in the wavelength of peak sensitivity (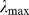) of the L and M cones; individuals possessing L or M opsins with atypical spectral sensitivities are referred to as anomalous trichromats [21]. The existence of these variations motivated us to study how the ability to classify L and M cones depends on the L∶M cone ratio and on the separation between the 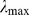 values of the L and M cones.

**Figure 4:**
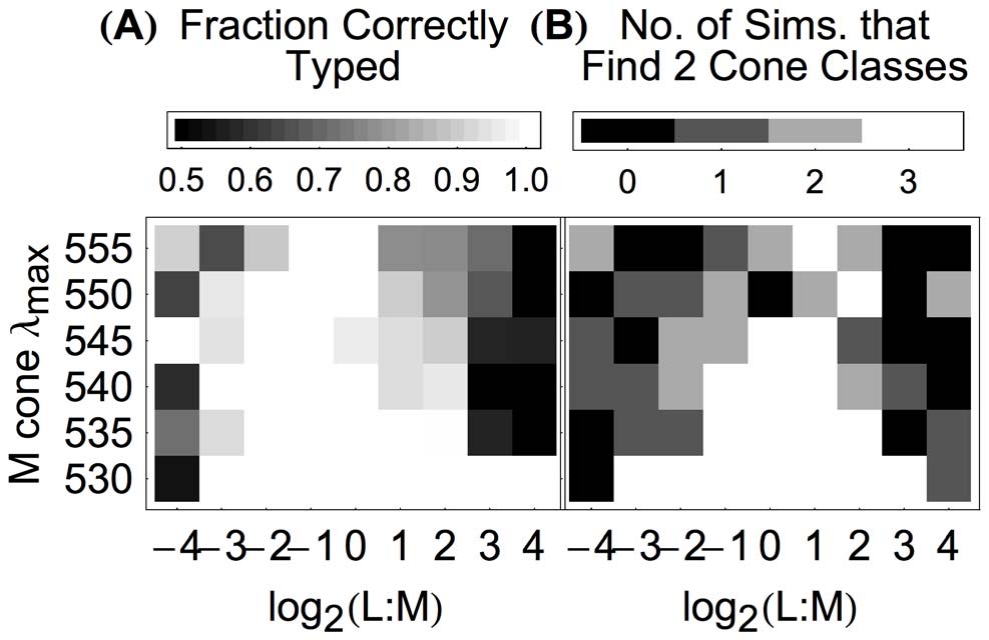
The algorithm detects the number of longer-wavelength-sensitive cone classes and correctly classifies individual cones for a range of trichromatic mosaic parameters. (**A**) The fraction of cones correctly classified for various combinations of L∶M cone ratio and M cone 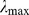 value, when the number of longer-wavelength cone classes (L and M) was assumed to be 2. The S cone proportion was held at 6%, and S cones were given a 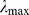 value of 420.7 nm in all simulations. The L cone 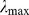 value was 558.9 nm. Each cell in the plot represents the aggregate results of three simulations, each with a different mosaic and each shown a different random sample of 2 million natural image patches. Mosaic responses for each simulation were obtained with different draws of natural image patches. Accuracies are reported as the average accuracy for L and M cones, each calculated separately. For example, if the algorithm correctly classified 354 of 354 L cones and 1 of 22 M cones in a mosaic with an L∶M ratio of 16∶1, the overall accuracy reported here would be 52% (the mean of 354/354 and 1/22) rather than 94% (the fraction of all cones correctly classified). (**B**) The number of simulations (of three) for each L∶M ratio and M cone 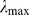 value where the algorithm correctly detected that there were two longer-wavelength-sensitive cone classes. The results for individual simulations are given in Figure S4.

Figure 4A shows the results. Each square in the figure indicates the mean percentage of cones correctly classified for a particular choice of L∶M cone ratio and particular separation of L and M 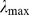 across three simulations. The results for individual simulations are given in Figure S4. Although both L and M cone 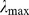 can vary across individuals, we parameterized differences by holding the L cone 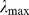 fixed at its typical value of 558.9 nm and varying the M cone 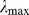 in 5 nm steps between its typical value of 530 nm and an upper bound of 555 nm. The figure shows that the algorithm is generally robust, but that classification performance falls off at the more extreme cone ratios. Approximately 16∶1 is the largest reported L∶M ratio for individuals whose color vision is normal by standard tests [20]. Algorithm performance also degrades with respect to decreases in the 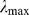 separation between the L and M cones.

The fact that good classification is accomplished with a 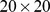 mosaic indicates that the information necessary to support learning can be accessed by local computations in either the retina or early retinotopic visual cortex. Indeed, we found that good learning was maintained when the mosaic size was reduced to 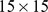 and was reduced only slightly for 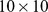 mosaics (Figure S5). In separate simulations, we examined the effect of showing fewer natural image patches to the retinal mosaic (Figure S6). For many of our mosaic conditions, asymptotic learning occurs for many fewer than the 2,000,000 images used in the main simulations.

Results for simulations without a suppressive surround are given in Figure S7. A comparison with Figure 4 shows that the presence of a randomly-wired surround enhances learning performance slightly relative to no surround while smaller retinal mosaics result in slightly worse learning. Additional simulations, run using a retinas with the same parameters as those shown in Figure 4 but such that the suppressive surround was cone selective (*i.e.*, L cone surrounds were made only of M cones, M cone surrounds were made only of L cones), were also performed (Figure S8). Interestingly, the presence of cone specific opponent surrounds early in the visual pathways impedes the performance of our algorithm.

**Figure 5:**
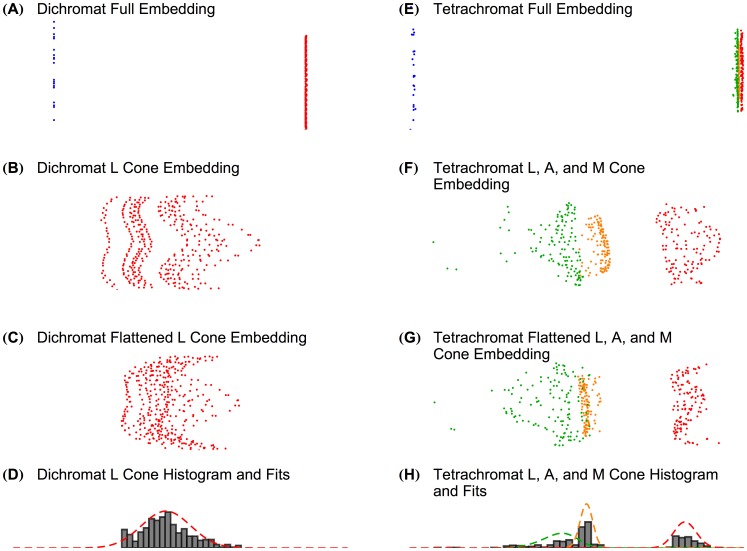
The algorithm detects dichromatic and tetrachromatic retinal mosaics. On the left are embeddings of the dichromatic retinal mosaic: (**A**) the full embedding; (**B**) the embedding zoomed in on just the L cones; (**C**) the flattened L cone embedding; and (**D**) a histogram of the 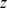 positions of the flattened L cone embedding with the best-fit of the single detected skew normal. On the right are embeddings of the tetrachromatic retinal mosaic: (**E**) the full embedding; (**F**) the embedding zoomed in on just the L, M, and anomalous (A) cones; (**G**) the flattened L, M, and A cone embedding; and (**H**) a histogram of the 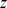 coordinates of the flattened L, M, and A cone embedding with best fit of a mixture of the detected skew normals. Note that the units on Panels **A**, **B**, **C**, **E**, **F**, and **G** are arbitrary, as MDS does not produce meaningful units, but rather yields a relative-distance-preserving embedding. Spectral sensitivity curves for L, M, and S cones are shown in Figure 2A. Anomalous A cones were given 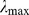 values of 545 nm, and the tetrachromatic retinal mosaic had an L∶M∶A ratio of 1∶1∶1. L, A, M, and S cones are colored red, yellow, green, and blue, respectively.

The algorithm as described and evaluated above relied on the assumption that the mosaic contains both L and M cones. This assumption entered the calculations at the step where we fit two skew normal distributions to the 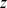 position histograms of the embedded cones (Figure 3D). In addition to variation in L and M cone 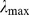, however, there are also individuals who are dichromats with mosaics containing S cones and only one longer-wavelength-sensitive cone class (*i.e.*, red/green color blindness). For such dichromatic retinal mosaics this algorithm would randomly divide the single longer-wavelength-sensitive cones into two classes. To address the question of whether it is possible to determine the number of longer-wavelength-sensitive cone classes present in a mosaic, we elaborated the algorithm with a step that compared how well a mixture of 1, 2, or 3 skew normal distributions fit the 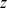 position histograms and chose the number of longer-wavelength-sensitive cone classes based on this fit. Specifically, we applied a Kolmogorov-Smirnov goodness of fit test [22] to the discovered mixture distributions and chose the smallest mixture number whose fit to the flattened 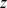 positions could not be rejected using a two-tailed critical 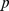-value of 0.01. When the elaborated algorithm is applied to the correlation matrices of a dichromatic retinal mosaic, it correctly detects that there is only a single longer-wavelength-sensitive cone class and thus classifies all of the longer-wavelength-sensitive cones as belonging together in a single class (Figure 5A–5D).

Given the success of the elaborated algorithm at correctly detecting that the dichromatic retinal mosaic is missing a cone class, we then asked whether it also detected that trichromatic retinal mosaics are in fact trichromatic. Figure 4B plots how well the elaborated algorithm detects that trichromatic retinal mosaics contain two classes of longer-wavelength-sensitive cones, as a function of L∶M ratio and 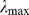 separation. Cells colored white indicate cases where the elaborated algorithm correctly detected the presence of both longer-wavelength-sensitive cone classes for all simulations. The pattern of accurate detection performance is generally similar to the patter of accurate classification performance, with detection accuracy falling off as the L∶M ratio becomes more extreme and as 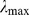 separation decreases. The range of good performance for detection shown, however, is narrower than the range of good classification. An examination of Figure S4 indicates that more cases of failure occur when the algorithm detects one (as opposed to three) longer-wavelength-sensitive cone classes than the other way around. Thus, we could presumably improve the range of good performance somewhat by adopting a more lenient criterion (lower critical 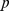-value) in our algorithm. We have not systematically explored this parameter space, however, as our view is that the key conclusion to be drawn from this work is that detection is possible for a reasonable range of mosaic parameters. A large number of additional factors (size of image patches, number of images sampled, presence of surround, cone-specificity of surround, nature of images sampled, optical blur, noisiness of cone responses) will all interact to push the exact boundaries of good performance as well as the direction of the failure modes around, and we do not wish to make too much of the particular location of these boundaries but rather focus on their general properties. Figures S5–S11 show both detection and classification performance as various of these factors are varied. Online supplemental material available at http://color.psych.upenn.edu/supplements/receptorlearning provides animated GIF renderings of the 3D embeddings corresponding to each of these supplemental figures (as well as to Figures S4 and S9). These additional simulations do reveal some cases where the algorithm detects three rather than two longer-wavelength-sensitive cone classes for some of the mosaic parameters. For the same reasons outlined above, we have not pursued the degree to which these over detections could be remedied by adjustment of the critical 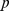-value or model selection procedure. Rather, the broad point we draw from the set of additional simulations is that the algorithm is generally robust; see Discussion for additional consideration of these simulations.

Some dichromats lack S cones but retain both L and M cones [23]. These individuals are called tritanopes and can make color discriminations that rely on longer-wavelength-sensitive cones. Accordingly, we simulated a set of tritanopic retinal mosaics containing only L and M cones. The resulting embeddings indicate that there is generally sufficient information in the inter-cone correlation matrix to classify the L and M cones (Figure S9; also online supplemental material referenced just above). As discussed in the legend of Figure S9, however, extracting this information automatically would require design and implementation of an additional algorithmic step that aligned the direction of cone separation in the embedding with the 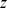 axis of our representational space, which we have not implemented. That is, the good alignment of the first dimension of the MDS solutions with the representational 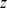 axis is driven by the presence of S cones in the mosaic, presumably because the presence of these cones produces greater correlation variation along this dimension (and in addition we could exploit the presence of S cones to refine the alignment). Additionally, simulation of a larger number of image patches (4.5 million) was required to produce good separation of the cone classes in the embedding. Thus, although the presence of S cones is probably not necessary for learning about longer-wavelength-sensitive cones, their presence does enhance the performance of our current algorithm. Note also that in the absence of S cones, the labeling of which cones are M and which are L becomes arbitrary; it is correct here because of the manual alignment procedure.

Some female humans possess genes that code for three longer-wavelength-sensitive cone photopigments, and there is evidence that all of these photopigments can be expressed in separate cones [24]. It is uncertain, however, whether these individuals process the output of their genetically tetrachromatic cone mosaics in a fashion that enables functional tetrachromatic color vision [25]–[27]. We simulated a tetrachromatic mosaic and applied our classification algorithm to the resulting correlation matrix. The fourth cone class, which we will call A, was given a 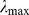 value of 545 nm, and the L∶M∶A ratio was 1∶1∶1. The algorithm correctly detected that this mosaic was tetrachromatic. Figure 5E–5H shows the classification results. The L, M, and A cones separate reasonably well but not perfectly in the embedding, and if we fit the flattened 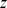 positions with three skew normal distributions (Figure 5H), the L, M, and A cones were classified with 100%, 100%, and 84% accuracy, respectively. This indicates that there is information in the correlation matrix that can support unsupervised cone classification in human tetrachromats, albeit imperfectly, at least for these specific simulation conditions.

In addition, we simulated tetrachromatic retinal mosaics with L, M, A, and S cone 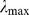 values of 558.9, 512.8, 466.8, and 420.7 (*i.e.*, with M and A cone 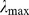 values evenly spaced between the typical L and S cone 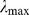 values) and with L∶M∶A ratios of 1∶1∶1, 2∶1∶1, 2∶2∶1, 1∶2∶1, 1∶2∶2, 1∶1∶2, and 2∶1∶2. We found that the algorithm correctly detected 3 longer-wavelength-sensitive cone classes in all cases and correctly classified all but 7 cones correctly between the 7 simulations.

## Discussion

The emergence of red-green color vision in primates is relatively recent, initiated by mutations that led to the expression of three rather than two cone opsins. In Old World Monkeys, this occurred approximately 35 million years ago via duplication of an ancestral M/L opsin gene followed by divergence of the two copies into the modern M and L cone opsin genes [1], [3], [4]. New World primates have also acquired functional trichromatic color vision, but through a different evolutionary path [2]. The recent evolution of the L/M split may mean that biochemical differentiation of post-receptoral circuitry has not yet occurred. Consistent with this, there are no known molecular markers that differentiate L and M cones aside from their opsins [5], [6]. Earlier work [7] established that unsupervised learning could produce a degree of cone-specific wiring, based on statistics of mosaic responses to natural images. Here we extend our understanding of what can be learned in two fundamental and important ways: we show that it is possible to learn the number of longer-wavelength-sensitive cone classes, and we quantify learning through the performance of an unsupervised learning algorithm that explicitly classifies each cone in the mosaic.

At present, the precise circuitry underlying red-green color vision in primates is not fully understood, and for this reason it is difficult to draw firm conclusions as to the locus where cone-class specific processing occurs. Some authors have suggested that circuitry in the adult retina does not distinguish L from M cones [28], [29]. They note that because midget cell centers in the primate fovea receive input from a single cone [30], random wiring of cones to ganglion cells would still preserve most of the color information in the fovea. This would then leave the full separation of L and M cone signals to be learned at the level of the cortex. Other authors [31]–[33] report varying degrees of L versus M cone-specific wiring for retinal ganglion cells. Such retinal specificity could result from activity-dependent synaptic plasticity during development [7], via the type of learning mechanisms considered here or in earlier work [7], [8].

For trichromatic mosaics, our algorithm correctly classified the cones in the retinal mosaic most of the time, with performance at ceiling for mosaic parameters most typical of human vision. The lowest accuracies are found when the L∶M ratio is either very large or very small. One reason for this is that our flattening routine performs best when the number of cones of each type is approximately equal (see Methods). This observation is equally true of the number of longer-wavelength-sensitive cone classes that our algorithm detects in the trichromatic simulations. The algorithm was most likely to detect only 1 longer-wavelength-sensitive cone class in the case of those simulations with extreme L∶M cone ratios. It is possible that a better flattening routine or an embedding algorithm more attuned to the correlation structure of natural images could lead to selective increases in our algorithm's accuracy for the more extreme L∶M ratios. On the other hand, it could be that the worsened flattening is a symptom reflecting a greater underlying difficulty in learning for mosaics with more extreme L∶M ratios. In this case, the fact that learning performance degrades as cone ratios become asymmetric may indicate fundamental constraints on the mosaic parameters that support trichromacy. The decreased performance with more extreme L∶M ratios was consistent across many variations in the details of the simulations (see Figures S4–S11).

Another subset of retinal parameters for which our algorithm's performance was degraded were the simulations whose M cone 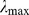 values were close to those of the L cones, primarily when the M cone 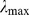 value was 555 nm, only 3.9 nm from the L cone 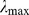 value of 558.9 nm. In most such cases, the algorithm grouped these cones into a single class. Considering the small difference in 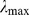, this is perhaps not surprising. Our results are thus consistent with experimental studies finding that intermediate color discrimination deficiencies result when the separation of L and M cone 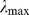 values is between 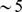
–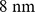 but severe color discrimination deficiencies when the L and M cone 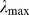 values are separated by 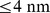 [21]. That said, the idea that learning limits performance in anomalous trichromats is speculative, as we have not attempted to quantify the performance losses that would be expected with a failure to correctly classify all of the cones. Nor have we compared this to the performance losses expected simply because a reduced separation in 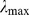 values reduces the signal-to-noise ratio of the information available to make color discriminations. Also of note with regard to the effect of 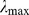 separation on performance is the fact that a single nucleotide polymorphism in the longer-wavelength-sensitive opsin gene can produce a shift in 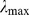 of 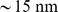 [34], so that a single mutational event could have produced a 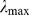 shift large enough to enable learning.

Performance for simulations that included a cone-specific surround (Figure S8) was lower than for simulations in which the surround drew uniformly on neighboring cones. Although the algorithm classified 100% of the cones correctly in the case of a retina with an L∶M ratio of 1 and an M cone 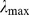 value of 530 nm, and also correctly detected that there were two longer-wavelength-sensitive cone classes present in the mosaic, the algorithm struggled with many other simulations with cone selective surrounds. In fact, the embeddings of these simulations were less organized than other simulations. Although a full analysis is beyond the scope of this paper, one feature of cone selective surrounds of fixed spatial extent in mosaics with extreme L∶M ratios is that the number of cones contributing to the surround will be different for units with L cone centers than for cones with M cone centers. It is possible that this produces variability that impedes learning.

Our algorithm correctly detected a tetrachromatic mosaic and did a reasonable but not perfect job of classifying the cones for this mosaic, indicating that there is sufficient information in the correlation of cone responses to natural images to support a degree of tetrachromatic learning. Although it is established that certain female carriers of anomalous trichromacy express four cone classes, whether these individuals exhibit tetrachromatic color matching performance has been difficult to determine [25]–[27]. As noted in the results section, our cone classification results were reasonable but not perfect, suggesting that one reason for the lack of routine functional tetrachromacy in female carriers may be that it is difficult for the post-receptoral visual system to reliably classify all of the cones [see also 7]. That is, it may be that rather narrowly delineated conditions must be met for the visual system to learn that its mosaic contains a fourth cone type and classify the cones with sufficient accuracy.

Dichromatic mosaics are also correctly detected by our learning algorithm (Figure 5A–5D), but more easily. This classification relies partly on an arbitrary choice of a confidence threshold (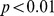) regarding the goodness-of-fit of the flattened 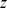 positions of the embedded correlation matrix to a single skew normal distribution, beneath which we reject dichromacy as an explanation of the retinal mosaic. Although this threshold is arbitrary, it represents a requirement that we be 99% confident that a distribution is not dichromatic in order to consider it trichromatic. Considering our algorithm's good performance on trichromatic mosaics, this implies that the dichromat/trichromat decision is one that the visual system could make reliably.

The multi-dimensional scaling approach that we took to explicitly classifying cones shares theoretical foundations with the approach taken in [7]. The point of commonality is that the coordinates of each cone on a dimension in an MDS representation may be viewed as analogous to the cone weights for a corresponding model unit obtained via principal components (PCA) or independent components (ICA) analysis. Indeed, cone positions obtained via classical metric MDS and cone weights obtained via PCA correspond to the rows and columns of the same underling matrix. That noted, different insights are obtained through the two approaches. Whereas in [7] the emphasis is most naturally on the degree of cone selectivity shown by the most selective model units, in our work the emphasis is on the accuracy of cone classification possible using the first three dimensions of the correlation matrix embedding. Indeed, the rotation and flattening of the embeddings performed by our algorithm use the information carried jointly by these first three dimensions. We have observed that perfect cone classification is possible in the absence of high cone-selectivity in the implied cone weights of model units corresponding to these dimensions. On the other hand, our algorithm does not take advantage of cone selectivity present in units beyond the initial three returned by the MDS algorithm. Given these similarities and differences, it is of interest to compare the results of our work with those obtained in [7]. First, we find that good classification is possible using the correlation matrix without considering higher-order statistical structure, whereas [7] did not find high cone selectivity with PCA. We believe that the primary reason for this difference is as noted above, that our algorithm classifies based on information carried jointly by multiple dimensions. Other factors probably also come into play (e.g., size of mosaics and number of images simulated both of which were larger in our work). None-the-less, both approaches lead to the conclusion that learning is improved with the addition of blur and is inhibited by a decrease in the spectral separation between L and M cones. In addition, we find that learning is improved in the presence of an opponent surround for each cone that draws non-selectively on its neighbors, consistent with an analysis of the effect of such surrounds on correlations (see Figure 8 in [7]). On the other hand, we find that we can detect the presence of a third longer-wavelength-sensitive cone class, while their approach did not lead to learning of cone-selectivity with respect to a mosaic representative of human tetrachromats. Given the considerations discussed above, this is probably a difference in degree and criterion. We also studied additional factors and find that learning remains possible when cone spacing is increased relative to the underlying image data, remains possible when noise is added to the simulated cone responses, becomes more difficult in the presence of a cone-selective opponent surround, and becomes more difficult as the L∶M cone ratio becomes more extreme.

Our work embodies a number of simplifying assumptions. First, we simulated photoreceptors arranged in a rectilinear grid rather than a more realistic hexagonal grid in order to more easily align the photoreceptors with the rectilinear pixel grid of the database images. Nothing in the algorithm relies on rectilinearity, however. Second, since we have limited information about the optical quality, and spatial resolution of the cameras used to acquire the hyperspectral images, we rely on a scale invariance assumption about natural images to interpret the hyperspectral images as being matched to foveal resolution. We also do not know how noisy the camera responses were nor how small artifacts due to limited camera sensor bit depth might have perturbed our results. A full exploration of the effect of camera parameters awaits a hyperspectral image dataset acquired at a resolution matched to that of human vision with a fully characterized camera. We doubt, however, that the basic features of the spatial and spectral correlations in natural images would be so sensitive to camera parameters so as to change our basic conclusions. To explore this, we investigated the effect of increasing cone spacing relative to the image scale by a factor of 2 (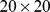 cone mosaic) and a factor of 4 (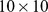 cone mosaic), for the standard L and M cone spectral sensitivities (558.9 and 530 nm) and an L∶M ratio of 1∶1. Learning continued to work well (2 classes of longer-wavelength-sensitive cones correctly detected and cones classed with 100% accuracy). This result suggests that our main analysis is not highly sensitive to image scale. It also serves to explore how learning might generalize from foveal to peripheral cones, since a key difference between fovea and periphery is an increase in cone spacing. Third, we assumed that the hyperspectral images in our database are representative of natural scenes. As a check on this assumption, we scrubbed the database of images of obviously man-made objects and repeated our simulations. The results are generally similar to those run using the full database, although there are more instances of failure to detect the correct number of longer-wavelength-sensitive cones (Figure S10). Finally, we assume that the responses of the cones to natural images contain no noise. To address this assumption, we performed simulations in which noise on the order of both 1% and 5% of the mean of the cone responses to each natural image was added randomly to each cone (Figure S11). The accuracy of the algorithm for these simulations is comparable with the corresponding no-noise results shown in Figure 4.

We did not incorporate the blur introduced by the optics of the human eye into our main simulations, in part because we do not know how much blurring was already introduced by the various hyperspectral cameras. In this regard, we note that the addition of optical blur makes the classification problem easier rather than harder for our algorithm (Figure S12). This is because blur reduces the variation in cone correlation caused by variation in the spatial structure of natural images [7]. It is therefore perhaps of interest that in humans, red-green color vision develops over the first three months of infancy [7], [35], [36], during a period when the optical quality of the image is not yet as good as that in adults [37], [38]. As an explicit test we explored the effect of introducing Gaussian blur (standard deviation of 4 pixels). Accuracies for the simulations with blur were high and are shown in Figure S12. The algorithm chose 2 cone classes in 40 of 54 simulations and correctly typed all cones in 49 of 54 simulations performed with blur. In addition to evidence that red-green human color vision develops after birth, monkeys raised in an unusual environment in which the illumination was always one of four monochromatic lights displayed impaired color discrimination [39]. Our algorithm as currently implemented does not capture this effect: we found that when we ran the algorithm with input that simulated the deprivation experiment, it still correctly detected that the mosaic was trichromatic and correctly classified all of the cones. However, the embedding (not shown) produced by this simulation, while leading to good separation by cone class along the 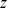 axis, was not typical of other simulation embeddings and did not produce an reasonable representation of the spatial positions of the cones as do our typical embeddings.

Why might the visual system have implemented a learning algorithm based on inter-cone correlations in advance of needing to determine the class of a newly expressed cone opsin? Previous authors [40], [41] have suggested that high acuity spatial vision requires learning of the spatial arrangement of cone positions, and suggested that spontaneous retinal activity as well as changes in interpolated neural images across eye movements provide information that would enable such learning. Although not the focus of this paper, our results (Figure S1) show that inter-cone correlations contain useful information for learning cone positions. Thus it seems possible that a learning algorithm based on this information could already have been in place as part of a system designed for spatial vision, and as such might have been ready to learn cone classes as well.

Another possibility is suggested by recent studies of adult dichromatic monkeys and of knock-in mice expressing the human long-wavelength opsin. Knock-in female mice heterozygous for the native mouse and human opsins have been shown to be capable of limited trichromatic discrimination [42]. Similarly, the addition of a third opsin using gene therapy can enable trichromatic color discriminations in adult dichromatic monkeys [43]. Although the enhanced discrimination does not necessarily mean that the visual system has used the new cone class to add a dimension to the perceptual representation of color [44], it does indicate that an immediate adaptive benefit can be bestowed by the expression of a novel photopigment, thus allowing for selective preservation of the mutation and subsequent enhancement through the implementation of a learning algorithm that classifies the cones.

Our work is at the computational level and does not speak directly to underlying neural mechanisms. Rather, we show that the information contained in the inter-cone correlation matrix in response to natural images is sufficient for detection of the number of cone classes as well as for classification of individual cones. Exploring how the information used by our algorithm might be exploited in a biologically-plausible manner, as well as how the learned information could be represented, remains an important future direction. For example, the computations described in [45] can be viewed as a method for using the knowledge of the class of each cone in the mosaic to determine the particular cone selective wiring that optimizes the veridicality of stimulus reconstruction, given a linking assumption of how the output of model units determined color appearance at each location. How the information used by algorithm could be used by a neural system to set up wiring with these particular properties, however, remains to be elucidated. Even in the absence of a neural theory, however, our work does make experimental predictions: altering inter-cone correlations during learning should alter how individual cones contribute to color vision. Recently it has become possible to independently stimulate individual primate cones using an *in vitro* retinal preparation [32] and similar possibilities with human psychophysics are on the horizon [46], [47]. Such techniques might be used to alter the correlation between a targeted M cone and its neighbors so as to test whether it is possible to make the post-receptoral visual system treat the responses from the targeted M cone as if they originated in an L cone. A second prediction of our work, to the extend that our current algorithm captures how the information available for learning varies with mosaic parameters, is that the range of observed L∶M cone ratios for human observers with functional trichromatic vision will decrease as the 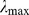 separation between the L and M cones decreases.

In terms of mechanism, it is also important to note that although the multidimensional scaling technique at the core of our algorithm provides one way to exploit the inter-cone correlations, we are not claiming that it is an optimal method nor that the neural representation of color includes an explicitly labeling of each type of cone. Multidimensional scaling is a well-established and principled unsupervised learning method that produces embeddings that allow detection of the number of cone classes and the classification of individual cones (Figure S3; online supplemental material). The components of our algorithm that exploit the structure of the embedding (flattening, fitting with skew normals, model comparison), however, are somewhat ad hoc and tailored to the specific problems we address. We view these steps as allowing us to quantify the quality of the information available in the embeddings, not as steps that necessarily reflect how a biological system would extract the same information. In any case, with respect to optimality, it is difficult if not impossible to evaluate our or any algorithm's optimality without an explicit probabilistic model of the statistical structure of natural images, and at present we do not have such a model [48]. Study of performance with respect to explicit models of natural image statistics would also allow investigation into what features of these statistics are key for supporting learning, a direction which we regard as interesting for future research. In addition, although we have shown that the inter-cone correlations contain sufficient information to enable the requisite learning, we have not shown that they are the only source of relevant information. For example, previous authors have suggested that comparison of interpolated neural images across eye movements might also provide information for separating L from M cones [8], [45]. Such a proposal is consistent with the idea that sensorimotor contingencies provide key information for enabling an organism to interact successfully with the external world [49].

With respect to representation, it may be that the the analysis of signals to extract an appropriate representational structure for stimuli, which is what MDS accomplishes when applied to response correlation matrices, is a canonical biological computation. If so, then the biological extraction of the information about cone classes that we demonstrate is available may occur, in effect, as a result of a more general perceptual learning processes. This notion also provides another possible explanation of why the brain is prepared to learn about and exploit the addition of novel classes of sensory transducers.

In summary, we have studied a question that is fundamental to the formation of any sensory system that provides multidimensional information. Given that the enabling step in the evolution of such systems must be the expression of novel sensory transducers, how is it possible to detect and differentiate their presence? We address these two fundamental questions at the computational level, using primate color vision as a model system. We find that inter-cone correlations provide sufficient information for unsupervised learning to detect the number of cone classes present and to classify individual cones accurately by demonstrating an algorithm that capitalizes on this information. These results fill an important gap in our understanding of how color vision could have evolved, and may also provide insight into the evolution and operation of other sensory systems.

## Methods

### Simulating cone responses

We simulated the responses of retinal mosaics to hyperspectral images. Individual mosaics were specified by 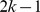 parameters: 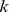, the number of cone classes in the simulated mosaic; 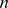, the size of the mosaic; 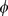, a 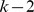 dimensional vector of the values of 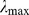 for cone classes other than L and S, whose 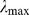 values were held at 558.9 and 420.7 nm respectively; and 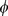, a 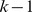 dimensional vector that determined the relative number of cones of different classes (see below). The code used to simulate cone responses and compute correlation matrices was developed in Clojure (http://clojure.org/), a functional open-source Java Virtual Machine language, and is freely available on gitHub (https://github.com/DavidBrainard/ReceptorLearning/).

Given mosaic size 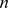, we simulated mosaics consisting of 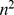 cones, with one cone at each location of an 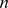 by 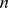 rectangular grid. The use of a rectangular grid was chosen for computational convenience. We specified that 6% of the cones be S cones; these were placed at quasi-regular locations by randomly choosing each S cone's position on the grid and rejecting it if it was too close to another already-placed S cone. The classes of the other cones in the mosaic were set randomly using the vector 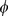 as a ratio in which the probability of drawing a cone of class 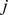 is equal to 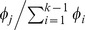; for example, an L∶M ratio of 4∶1 can be expressed by 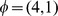 such that the probability of drawing an L cone would be 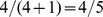 and the probability of drawing an M cone would be 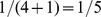.

Given the class of a cone, we determined its spectral sensitivity from its photopigment's wavelength of maximum sensitivity 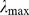. As noted above, 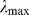 values for L and S cones were held at 558.9 and 420.7 nm respectively. These correspond to typical values for a human observer. Across simulations, we varied 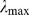 for the M cones between its typical value of 530 nm up to values of 555 nm to simulate anomalous trichromacy. To simulate human tetrachromatic mosaics, we specified an M cone 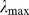 of 530 nm and 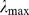 for a fourth cone type of 545 nm. Given its 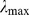 value, the spectral sensitivity of a cone was determined using the CIE 2-degree physiological cone fundamental standard [50], as implemented in the Psychophysics Toolbox [51]. This implementation extends the CIE standard by allowing specification of non-standard 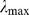 values via the photopigment absorbance nomogram of Stockman *et al.* [52]. Figure 2A shows an example mosaic; Figure 2B shows spectral sensitivities for typical L, M, and S cones (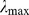 values of 558.9, 530, and 420.7 nm, respectively). This same procedure was also used in other simulation conditions where 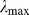 was varied.

Input images for simulations were taken from three hyperspectral image databases, details of each of which can be found elsewhere [10]–[12]. The images in all three databases sampled scenes along at least 31 evenly-spaced wavelengths. The sampling depended on the database. All contained data between 420 and 700 nm at 10 nm steps; across databases we had data between 400 nm and 720 nm. Of the 33 total wavelengths available across all three databases, we extrapolated quadratically to the missing two (either 400 and 410 nm or 710 and 720 nm) when necessary so that the combined database specified the spectrum at each pixel in each image at 10 nm intervals between 400 and 720 nm. Although there were fewer than 100 images in the three databases, the simulated mosaics were sufficiently small and the images sufficiently large that we were able to sub-sample the full images to produce many possible image patches. For each simulation, 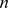 by 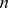 image patches were randomly chosen from the image database. For the value of 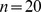 used in our main simulations, the database contained 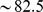 million distinct image patches with overlap allowed. Unless otherwise specified, each simulation used a different set of natural image draws. Image patches were drawn randomly with replacement. For our main calculations, we simulated the response of each specified mosaic to 2 million image patches. In supplemental simulations, we explored the effect of varying the number of image patches used (Figure S6) as well as the size of the patches (Figure S5).

To simulate the response of a mosaic to an image, we first computed the raw response 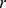 of each mosaic cone to the image. This was calculated as 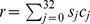 where 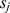 and 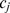 are the spectral sensitivity of the photoreceptor and the image intensity, respectively, at wavelength 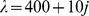 (in nanometers). This gave us the raw responses to the image that are proportional to photopigment isomerization rates. Such rates, however, are not directly available to post-receptoral circuitry, because lateral processes in the retina form an antagonistic surround that may be measured as early as the cone synapse [13], [14]. To capture this effect, we computed a suppressive surround for each cone by computing a weighted sum of the raw responses of the cone's neighbors, with the weights 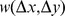 chosen according to a 2D Gaussian in which 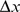 and 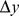 represent the distance from the cone of interest to its neighbors in units of inter-cone distance. The surround value was subtracted from the cone response to provide the signal available to our learning algorithm. We used a standard deviation of 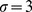 cones and 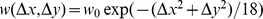) where 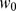 was a constant chosen to give the surround a sum of 0.25. In supplemental simulations, we simulated mosaics without a surround (Figure S7) as well as mosaics with a cone-specific surround in which L cone surrounds were composed only of M cones and vice versa (Figure S8).

### Analysis of responses

The responses of each cone from a particular set of natural images were used to calculate an 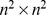 correlation matrix 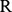. We used 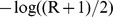 as an approximation of the distance between cones in the 3D representational space of 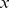, 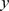, and 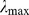, *i.e.*, position on the retina and cone class. We applied non-metric multidimensional scaling (MDS) to this distance metric to produce a 3D embedding of the response correlations. MDS was performed using the mdscale function in Matlab 8.1.0.604 (R2013a). A three dimensional solution was requested and all parameters were given default values. Specifically, the following default parameters were used: ‘Criterion’: ‘stress’ (also known as STRESS1); ‘Start’: ‘cmdscale’ (use classical multidimensional scaling as the starting state); ‘Replicates’: 1; ‘Weights’: uniform (*i.e.*, all weights were 1). Figure 3A shows the 3D embedding of the mosaic shown in Figure 2A, based on 2 million images. We consistently found (for 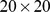 mosaics that contained S cones) that the first dimension of the MDS solution corresponded approximately to the 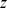 (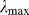) dimension of our desired representational space. This alignment was refined by rotating embeddings such that the separation between non-S cone and S cone positions fell along the first dimension by aligning the vector pointing from the mean of the longer-wavelength-sensitive cone positions to the mean of the S cone positions to the vector 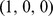. For most simulations, this rotation was negligible, with the exception being 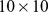 and 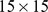 mosaics. In our algorithm, we used 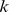-means clustering with 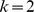 to identify the S cones. These were taken as the members of the cluster with fewer cones. In the tritanope simulations, this step was skipped, and instead embeddings were rotated by hand to resemble the orientation of other non-tritanope simulations, using knowledge of which cones were M and which were L.

The L and M cone embeddings were flattened by modeling the average surface along which the embedded L and M cones fell (see, for example, Figure S3), and removing the curvature of that modeled surface from the cones in the embedding. This step, as well as the fitting of skew normals and model comparison, was implemented in Mathematica, and the implementation's code is also available in our gitHub repository (https://github.com/DavidBrainard/ReceptorLearning/). The model surface was discovered by minimizing the standard deviation of the distance from each non-S cone coordinate in the embedding to a second order polynomial surface parameterized by the second and third embedded dimensions (*i.e.*, 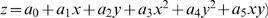). The flattened 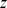 value was calculated as the distance of each cones unflattened embedded position to the surface; the sign of the flattened 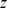 value was negative if the point was on the same side of the surface as the S cones and was positive if the point was on the other side. (For the tritanopic simulations, this direction was determined manually as part of the embedding rotation procedure described above.) The standard deviation of the distances from the surface was chosen as the function to be minimized; this encourages the nonlinear minimization to find the surface that is equally distant from each cone rather than a surface that is close to most cones. When the sum of residual distances is minimized instead, the discovered surface can sometimes find an optimal fit without capturing the shape of the surfaces on which the cones are embedded. Notably, these minimization techniques perform best when the numbers of cones in each class are approximately the same. In the cases where the L∶M cone ratios are extreme, the surface can over-represent the more numerous cone class, resulting in a flattened embedding in which the more numerous cone class has been flattened, but the other cone class is spread over a large range in the 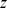 dimension.

The flattened 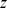 positions were used to classify cone types. A mixture distribution of 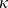 skew normal distributions was fit to the flattened 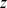 positions using nonlinear minimization. For each 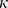, a two-tailed 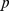-value of the Kolmogorov-Smirnov goodness-of-fit [22] was calculated such that the 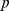-value gave the probability that the flattened 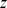 positions came from the fit distribution. This 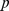-value was used to discriminate between possible choices of 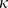. The distribution with the fewest parameters (*i.e.*, the smallest number of predicted cone classes) whose 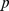-value was 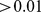 was selected as the best fit. Cones were divided into 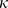 classes according to the distribution in the mixture whose value was highest at that point's flattened 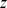 position. Skew normal distributions were assumed to represent one cone class each and were assumed to represent cones with higher 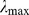 values when their mean 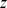 positions were farther from those of the S cones.

## Supporting Information

Figure S1
**The algorithm finds the approximate relative spatial locations of the L and M cones.** The figure shows the arrangement of the points in the 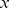-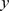 plane for our algorithm's embedding of the randomly-generated retinal mosaic shown in Figure 2A. The original (correct) mosaic is plotted as open circles colored red and green for L and M cones according to their identity in the mosaic. The embedding is plotted as filled points, colored according to the cone class found by the algorithm. Lines are drawn from the embedded position of each cone to its correct mosaic position. A rigid least-squares relative-distance-preserving transformation in the 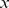-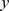 plane was applied to align the embedded positions to the original (correct) locations prior to plotting. Note that 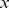 and 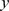 here refer to the representational space discussed in the Results section and not to the first two dimensions of the raw MDS solution.(TIFF)

Figure S2 **The distances in the multidimensional scaling embedding capture the structure of the correlation matrix.** The figure shows the probability density function of the correlation between pairs of cones during the simulation versus their distance in the 3D embedding. This figure is for the simulation whose mosaic is shown in Figure 2. Each contour line represents approximately 4,725 pairs of cones.
(TIFF)

Figure S3
**The 3D embeddings of the L and M cones vary systematically with changes in L∶M ratio and** 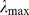 **separation.** Example embeddings are shown for L∶M ratios varying from 1∶16 to 16∶1 and for M cone 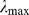 values ranging from 530 nm 555 nm; results for these conditions are also shown in Figure 4 and are arranged identically here. In many cases, the L and M cones appear as 2D surfaces with similar shape but with different offsets. Rotating animations that show the three-dimensional structure of these embeddings are available online at: http://color.psych.upenn.edu/supplements/receptorlearning.(TIFF)

Figure S4
**Algorithm performance is consistent across individual runs of the simulations.** This figure shows the fraction of cones correctly classified in each of the three simulations that were aggregated to produce Figure 4. The left column shows the fraction of cones correctly classified for various combinations of L∶M ratio and M cone 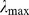 value, when the number of longer-wavelength-sensitive cone classes was assumed to be 2. The right column shows the number of longer-wavelength-sensitive cone classes (1, 2, or 3) detected by the algorithm for each L∶M ratio and M cone 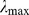 value. All three simulations were run with different randomly-generated retinal mosaics and different draws of 2 million natural image patches. Reported accuracies in the left column are the average of the fraction of L and M cones correctly typed.(TIFF)

Figure S5
**Algorithm performance varies across image patch size.** As the size of the image patch declines, classification performance remains stable, while detection performance declines slightly. All retinal mosaics were shown a different set of 2 million randomly-drawn natural image patches. (**A**) The fraction of cones correctly typed for a 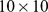 mosaic (left) and a 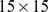 mosaic (right), for various combinations of L∶M ratio and M cone 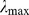 value, when the number of longer-wavelength-sensitive cone classes was assumed to be 2. S cones were held at 6% of the cones and were given a 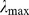 value of 420.7 in all simulations. (**B**) The number of longer-wavelength-sensitive cone classes detected by the algorithm for each L∶M ratio and M cone 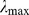 value.(TIFF)

Figure S6
**Algorithm performance depends on the number of simulated image patches.** The figure panels show the accuracy of the classification algorithm as a function of the number of natural image patches shown to the mosaic for simulations in which the M cone 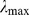 was 530 nm (bottom), 540 nm (middle), and 550 nm (top). The number of images required to achieve good classification varies with L∶M ratio and 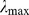 separation, but reaches asymptotic levels at about 200,000–300,000 images for retinas with an M cone 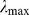 value of 530 nm or 540 nm and an L∶M ratio of 1. Simulations with an L∶M ratio of 1 but with an M cone 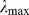 value of 550 nm have high performance but the performance is less stable. Although 300,000 images may seem like a large number, it represents fewer than one image per second for 6 hours per day for two weeks. Classification accuracy is lower and less stable for mosaics with M cone 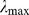 values near that of the L cones and mosaics whose L∶M ratios are much greater than or less than 1. Because our entire natural image database was several gigabytes in size, it was much more efficient to read each natural image only once during a simulation and to draw all patches from the simulation in batch. Accordingly, the ordering of the images is not fully shuffled for numbers of image patches less than 2,000,000 (our sampling algorithm does ensure equal representation for the primary case of 2,000,000 image patches studied in the main conditions of the paper.) The non-random sampling may be responsible for the fluctuations in performance with image number that can be seen for some simulation conditions.(TIFF)

Figure S7
**Algorithm performance degrades somewhat without simulated surround suppression.** This figure shows the fraction of cones correctly classified when no surround suppression was simulated. The performance of the algorithm, when compared to Figure 4, degrades most clearly as the M and L cone 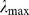 values approach each other, suggesting that surround suppression helps distinguish cones when their responses are very similar. All simulations were shown a different randomly-drawn set of 2 million hyperspectral image patches. (**A**) Fraction of cones correctly classified for various combinations of L∶M ratio and M cone 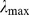 value, when the number of longer-wavelength-sensitive cone classes was assumed to be 2. S cones were held at 6% of the cones and were given a 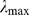 value of 420.7 in all simulations. Accuracies are the average of the fraction of L and M cone correctly typed. (**B**) The number of longer-wavelength-sensitive cone classes detected by the algorithm for each L∶M ratio and M cone 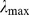 value.(TIFF)

Figure S8
**The algorithm performs more poorly when a cone-specific surround suppression is employed.** Simulations were run with a Gaussian surround suppression (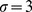 cones, weight = 0.25) such that each cone was suppressed only by nearby cones of different type. L and M cones opposed each other, and S cone surrounds consisted of both L and M cones. In the case of retinas with extreme L∶M ratios, this often required that some cones have no surround (no nearby cones of a different type) while others had very strong surrounds (all nearby cones were of a different type). Each retinal mosaic was shown a different set of 2 million randomly-drawn natural image patches. (**A**) The fraction of cones correctly typed for a 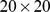 mosaic, for L∶M ratios ranging from 16∶1 to 1∶16 and for M cone 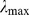 values ranging from 530 nm and 555 nm. The number of cone classes was assumed to be 2. S cones were held at 6% of the cones and were given a 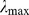 value of 420.7 in all simulations. (**B**) The number of longer-wavelength-sensitive cone classes detected by the algorithm for each L∶M ratio and M cone 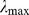.(TIFF)

Figure S9
**Information in the correlation matrix supports classification of L and M cones for tritanopes (no S cones).** Results are shown for simulations of tritanopic mosaics with 4.5 million natural image patches. This is more image patches than were required for mosaics containing S cones. Although separation of L and M cones could be clearly observed in the 3D embeddings with 4.5 million image patches (see online supplement), the embeddings produced by the MDS algorithm were not aligned such that the first dimension of the MDS solution corresponded to the direction along which L and M cones separated. Nor, obviously, could we leverage the positions of the S cones to rotate the solution to produce such alignment as we did for simulations of mosaics with S cones. Accordingly, we manually rotated these embeddings before applying the flattening and classification steps of our algorithm so as to be able to quantify the separation between the L and M cones that could be achieved in a manner comparable with our other simulations. (**A**) The fraction of cones correctly typed for a 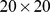 mosaic, for various values of the L∶M ratio when the number of cone classes was assumed to be 2. The 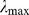 values for L and M cones were 558.9 and 530.3 nm, respectively. Note, however, the labeling of which cones are L and which are M by our algorithm becomes arbitrary in the absence of an S cone anchor. This arbitrariness was accounted for here as part of the manual rotation of the embedding. (**B**) The number of longer-wavelength-sensitive cone classes detected by the algorithm for each L∶M ratio.(TIFF)

Figure S10
**The algorithm performs well for images that exclude man-made objects.** Performance is similar to those simulations in Figure 4, in which man-made items were not scrubbed from the image set, although there are a set of cases for M cone-rich retinas where three rather than two longer-wavelength-sensitive cone classes are detected. All retinal mosaics were shown a different set of 2 million randomly-drawn natural image patches. (**A**) The fraction of cones correctly typed for a 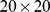 mosaic, for various combinations of L∶M ratio and M cone 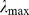 value, when the number of longer-wavelength-sensitive cone classes was assumed to be 2. S cones were held at 6% of the cones and were given a 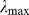 value of 420.7 in all simulations. (**B**) The number of longer-wavelength-sensitive cone classes detected by the algorithm for each L∶M ratio and M cone 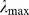 value.(TIFF)

Figure S11
**The algorithm is robust to noise.** Noise was modeled by adding to each cone's response a random draw from a normal distribution whose standard deviation was 1% (left) or 5% (right) of the mean response from all cones. Each retinal mosaic was shown a different set of 2 million randomly-drawn natural image patches. (**A**) The fraction of cones correctly typed for a 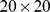 mosaic, for L∶M ratios ranging from 16∶1 to 1∶16 and for M cone 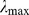 values ranging from 530 nm and 555 nm. The number of cone classes was assumed to be 2. S cones were held at 6% of the cones and were given a 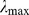 value of 420.7 in all simulations. (**B**) The number of longer-wavelength-sensitive cone classes detected by the algorithm for each L∶M ratio and M cone 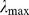.(TIFF)

Figure S12
**The algorithm performs well when images are blurred**, although there are a few cases where three rather than two longer-wavelength-sensitive cone classes are detected. Blurring was accomplished by convolving each image with a 2D Gaussian with a standard deviation of 4 pixels. Each retinal mosaic was shown a different draw of 2 million natural image patches. (**A**) The fraction of cones correctly typed for a 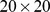 mosaic, for various combinations of L∶M ratio and M cone 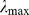 value, when the number of longer-wavelength-sensitive cone classes was assumed to be 2. S cones were held at 6% of the cones and were given a 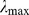 value of 420.7 in all simulations. (**B**) The number of longer-wavelength-sensitive cone classes detected by the algorithm for each L∶M ratio and M cone 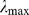 value.(TIFF)
