# Supplementary figures and images for: Unsupervised Learning of Cone Spectral Classes from Natural Images
Source: PLoS Comput Biol. 2014 Jun 26;10(6):e1003652. doi: 10.1371/journal.pcbi.1003652 (PMC4072515; doi:10.1371/journal.pcbi.1003652)

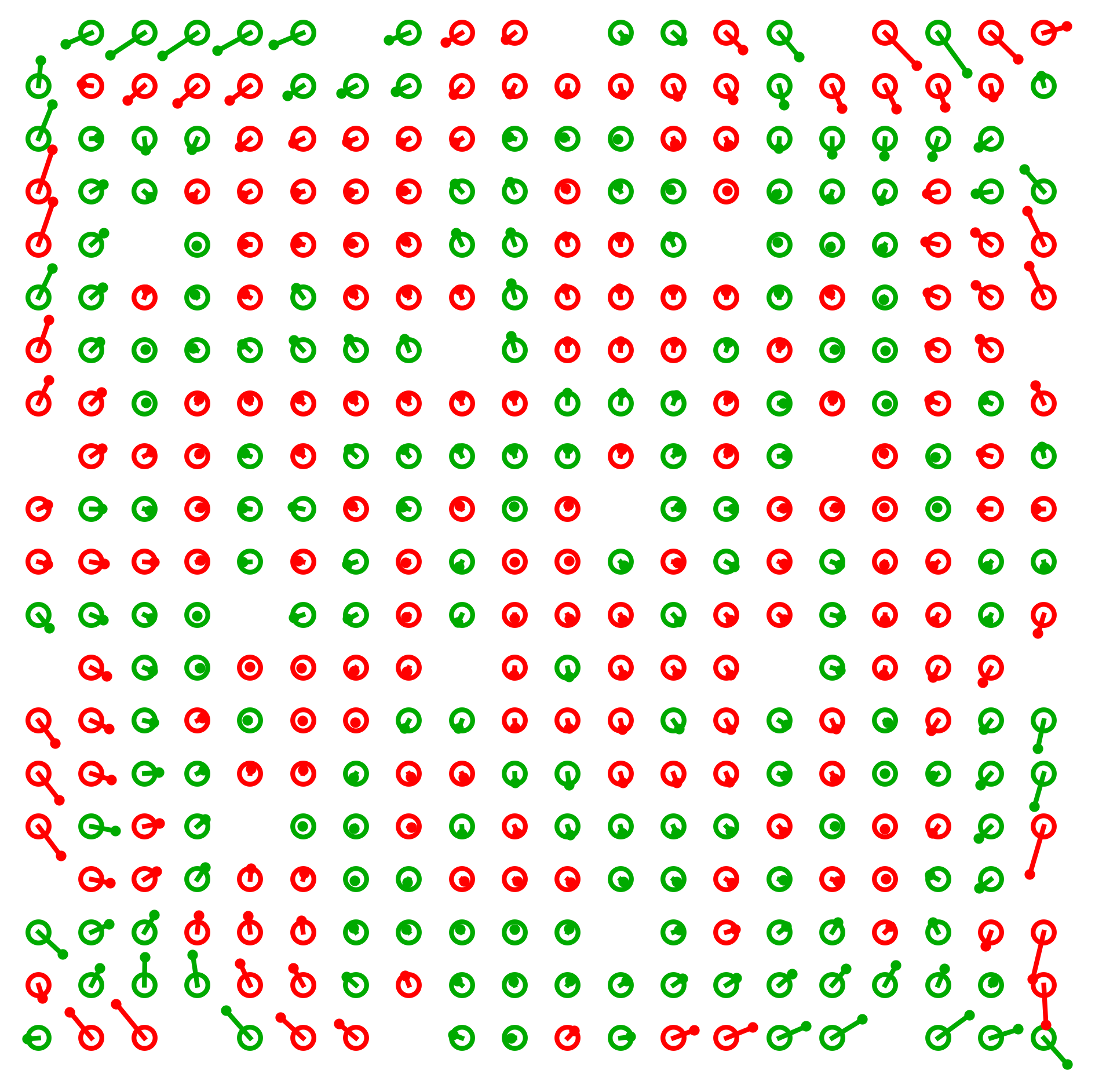

Supplement: Figure S1 — The algorithm finds the approximate relative spatial locations of the L and M cones. The figure shows the arrangement of the points in the - plane for our algorithm's embedding of the randomly-generated retinal mosaic shown in Figure 2A. The original (correct) mosaic is plotted as open circles colored red and green for L and M cones according to their identity in the mosaic. The embedding is plotted as filled points, colored according to the cone class found by the algorithm. Lines are drawn from the embedded position of each cone to its correct mosaic position. A rigid least-squares relative-distance-preserving transformation in the - plane was applied to align the embedded positions to the original (correct) locations prior to plotting. Note that and here refer to the representational space discussed in the Results section and not to the first two dimensions of the raw MDS solution. (TIFF) [file pcbi.1003652.s001.tiff]

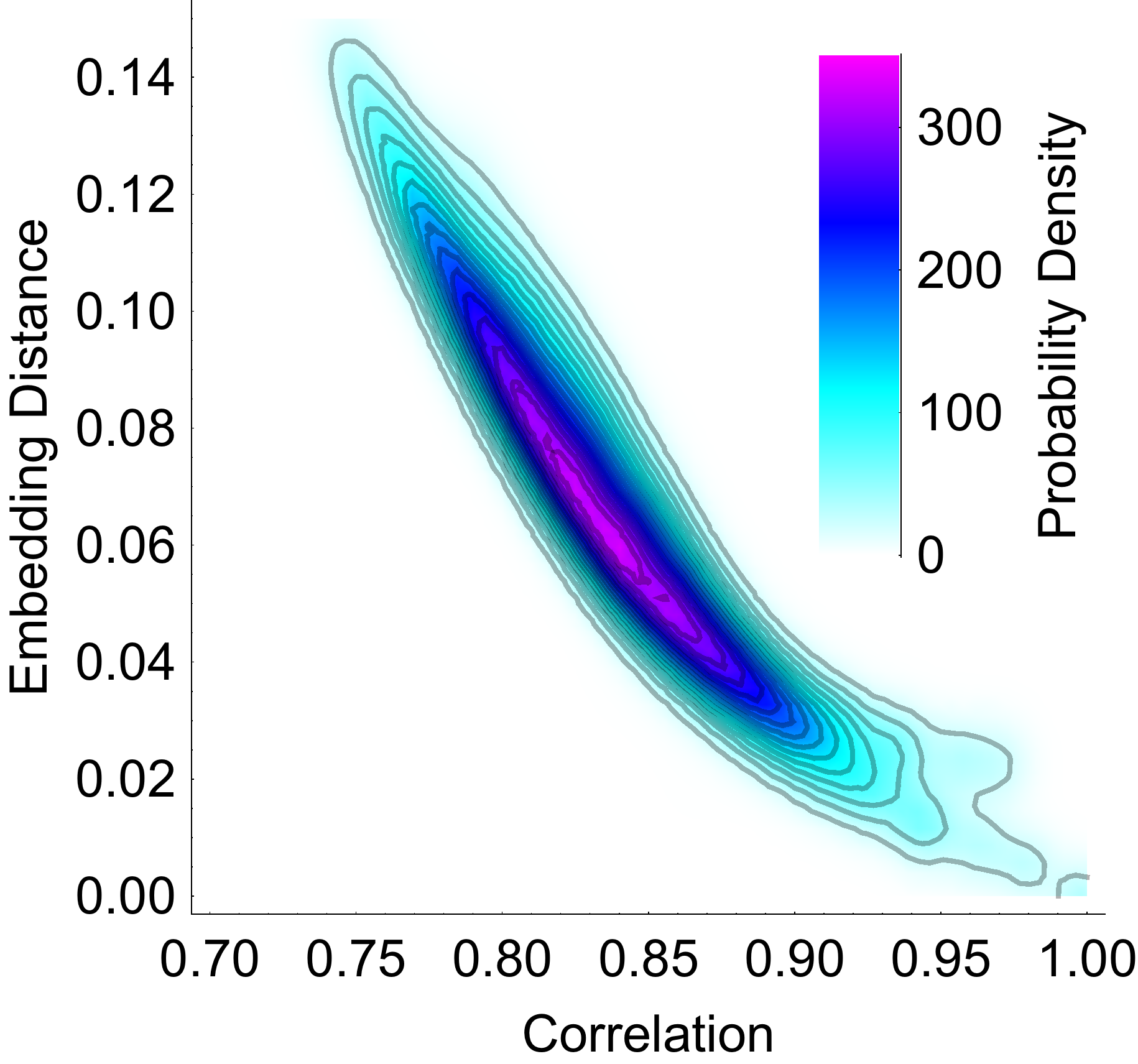

Supplement: Figure S2 — The distances in the multidimensional scaling embedding capture the structure of the correlation matrix. The figure shows the probability density function of the correlation between pairs of cones during the simulation versus their distance in the 3D embedding. This figure is for the simulation whose mosaic is shown in Figure 2. Each contour line represents approximately 4,725 pairs of cones. (TIFF) [file pcbi.1003652.s002.tiff]

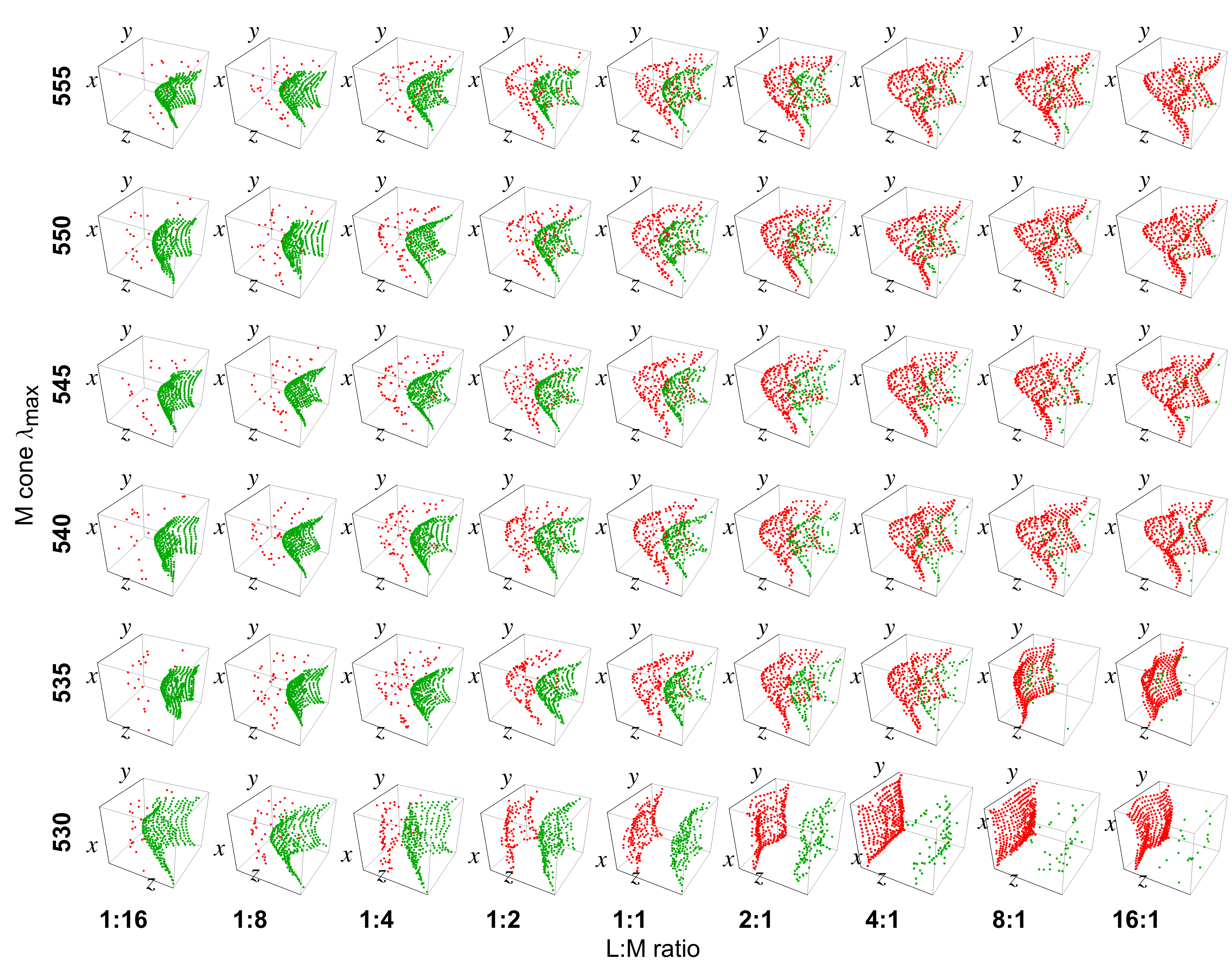

Supplement: Figure S3 — The 3D embeddings of the L and M cones vary systematically with changes in L∶M ratio and separation. Example embeddings are shown for L∶M ratios varying from 1∶16 to 16∶1 and for M cone values ranging from 530 nm 555 nm; results for these conditions are also shown in Figure 4 and are arranged identically here. In many cases, the L and M cones appear as 2D surfaces with similar shape but with different offsets. Rotating animations that show the three-dimensional structure of these embeddings are available online at: http://color.psych.upenn.edu/supplements/receptorlearning. (TIFF) [file pcbi.1003652.s003.tiff]

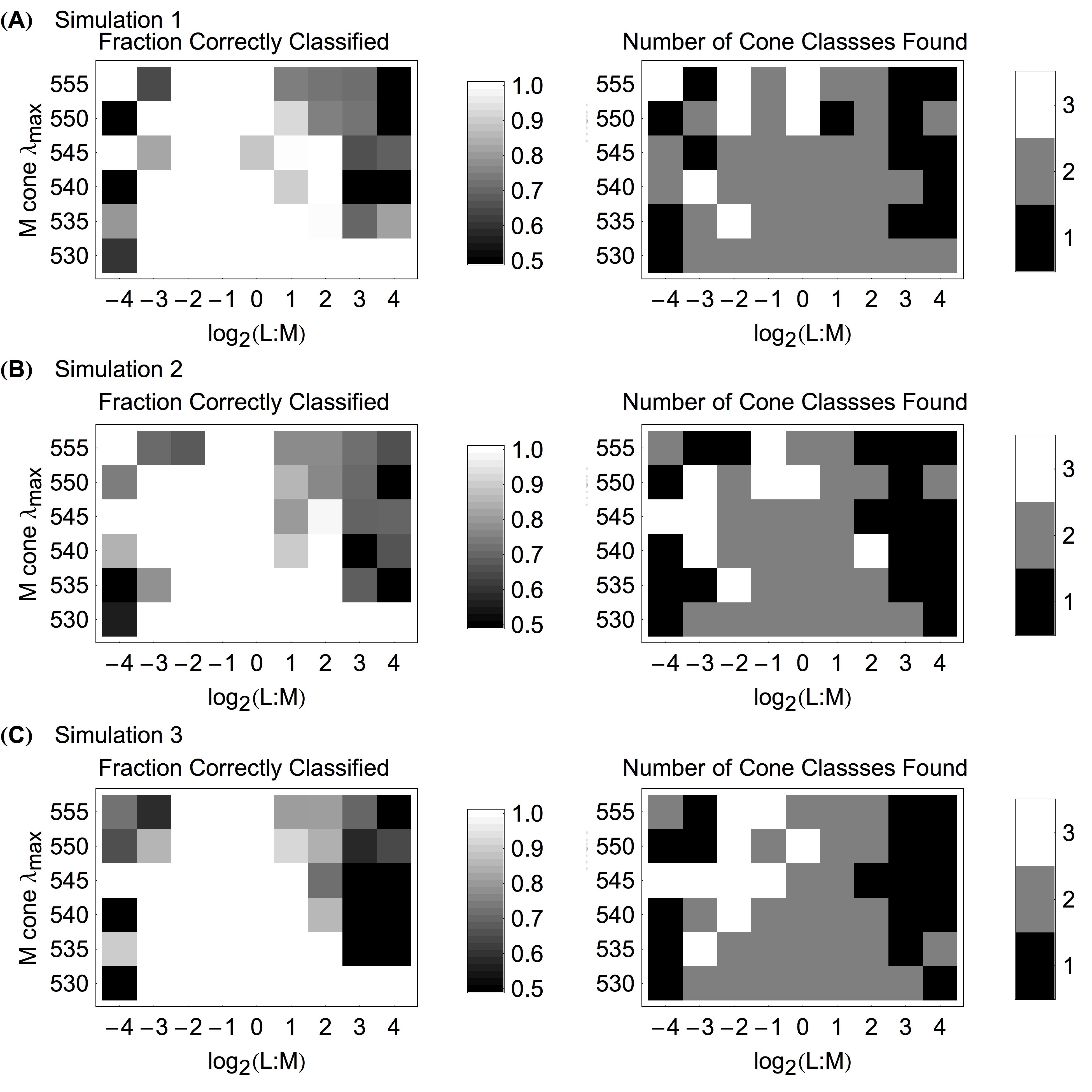

Supplement: Figure S4 — Algorithm performance is consistent across individual runs of the simulations. This figure shows the fraction of cones correctly classified in each of the three simulations that were aggregated to produce Figure 4. The left column shows the fraction of cones correctly classified for various combinations of L∶M ratio and M cone value, when the number of longer-wavelength-sensitive cone classes was assumed to be 2. The right column shows the number of longer-wavelength-sensitive cone classes (1, 2, or 3) detected by the algorithm for each L∶M ratio and M cone value. All three simulations were run with different randomly-generated retinal mosaics and different draws of 2 million natural image patches. Reported accuracies in the left column are the average of the fraction of L and M cones correctly typed. (TIFF) [file pcbi.1003652.s004.tiff]

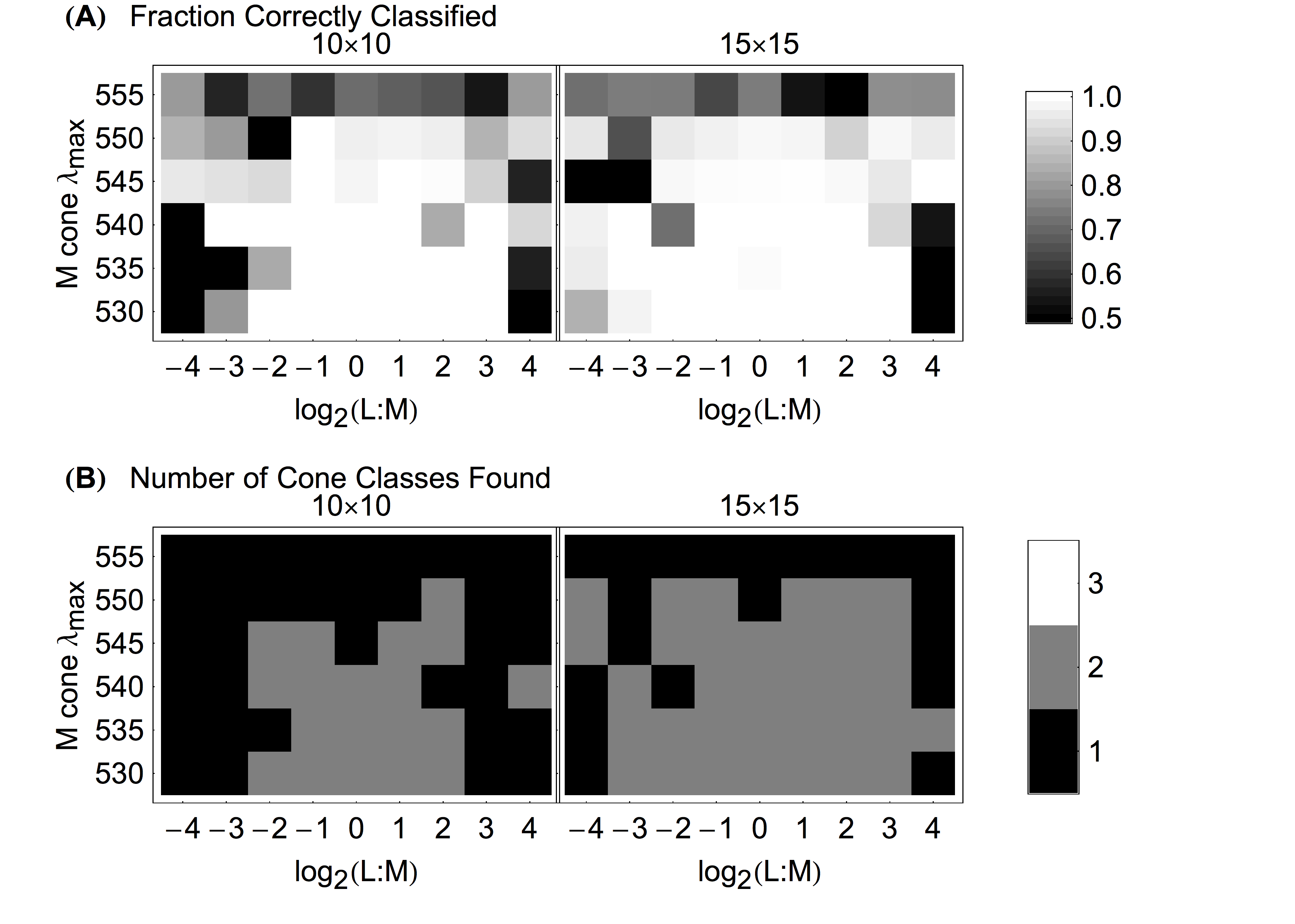

Supplement: Figure S5 — Algorithm performance varies across image patch size. As the size of the image patch declines, classification performance remains stable, while detection performance declines slightly. All retinal mosaics were shown a different set of 2 million randomly-drawn natural image patches. (A) The fraction of cones correctly typed for a mosaic (left) and a mosaic (right), for various combinations of L∶M ratio and M cone value, when the number of longer-wavelength-sensitive cone classes was assumed to be 2. S cones were held at 6% of the cones and were given a value of 420.7 in all simulations. (B) The number of longer-wavelength-sensitive cone classes detected by the algorithm for each L∶M ratio and M cone value. (TIFF) [file pcbi.1003652.s005.tiff]

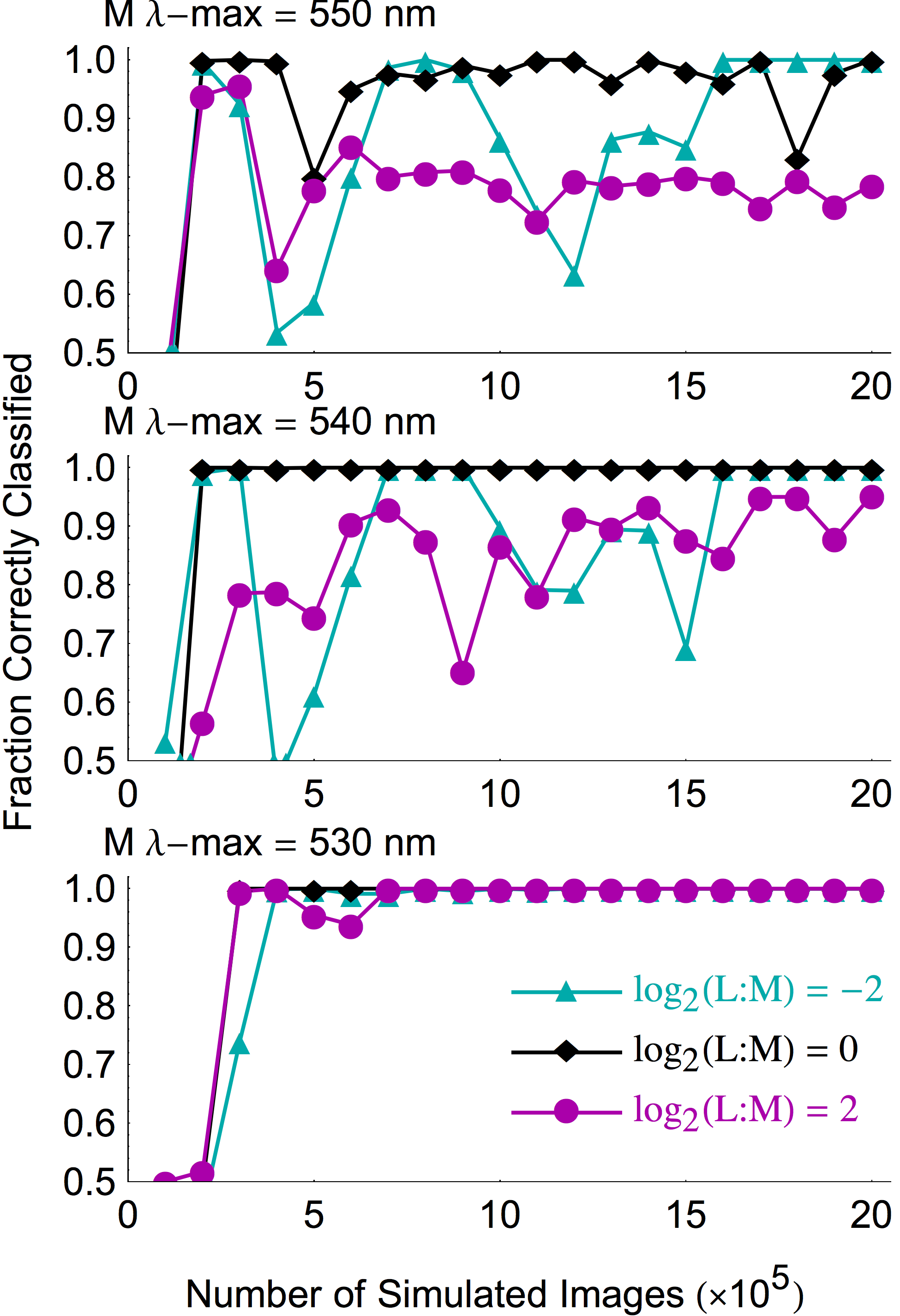

Supplement: Figure S6 — Algorithm performance depends on the number of simulated image patches. The figure panels show the accuracy of the classification algorithm as a function of the number of natural image patches shown to the mosaic for simulations in which the M cone was 530 nm (bottom), 540 nm (middle), and 550 nm (top). The number of images required to achieve good classification varies with L∶M ratio and separation, but reaches asymptotic levels at about 200,000–300,000 images for retinas with an M cone value of 530 nm or 540 nm and an L∶M ratio of 1. Simulations with an L∶M ratio of 1 but with an M cone value of 550 nm have high performance but the performance is less stable. Although 300,000 images may seem like a large number, it represents fewer than one image per second for 6 hours per day for two weeks. Classification accuracy is lower and less stable for mosaics with M cone values near that of the L cones and mosaics whose L∶M ratios are much greater than or less than 1. Because our entire natural image database was several gigabytes in size, it was much more efficient to read each natural image only once during a simulation and to draw all patches from the simulation in batch. Accordingly, the ordering of the images is not fully shuffled for numbers of image patches less than 2,000,000 (our sampling algorithm does ensure equal representation for the primary case of 2,000,000 image patches studied in the main conditions of the paper.) The non-random sampling may be responsible for the fluctuations in performance with image number that can be seen for some simulation conditions. (TIFF) [file pcbi.1003652.s006.tiff]

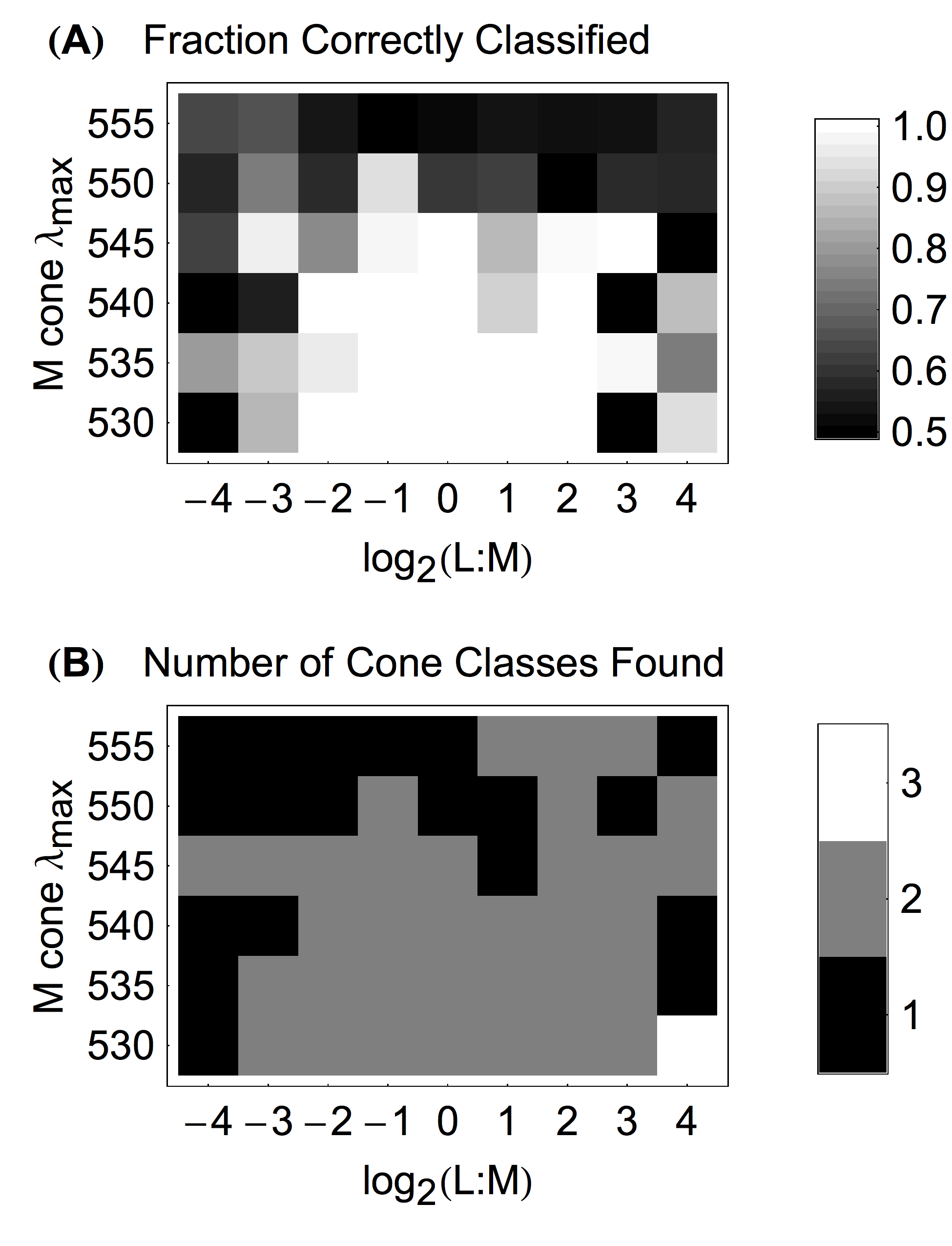

Supplement: Figure S7 — Algorithm performance degrades somewhat without simulated surround suppression. This figure shows the fraction of cones correctly classified when no surround suppression was simulated. The performance of the algorithm, when compared to Figure 4, degrades most clearly as the M and L cone values approach each other, suggesting that surround suppression helps distinguish cones when their responses are very similar. All simulations were shown a different randomly-drawn set of 2 million hyperspectral image patches. (A) Fraction of cones correctly classified for various combinations of L∶M ratio and M cone value, when the number of longer-wavelength-sensitive cone classes was assumed to be 2. S cones were held at 6% of the cones and were given a value of 420.7 in all simulations. Accuracies are the average of the fraction of L and M cone correctly typed. (B) The number of longer-wavelength-sensitive cone classes detected by the algorithm for each L∶M ratio and M cone value. (TIFF) [file pcbi.1003652.s007.tiff]

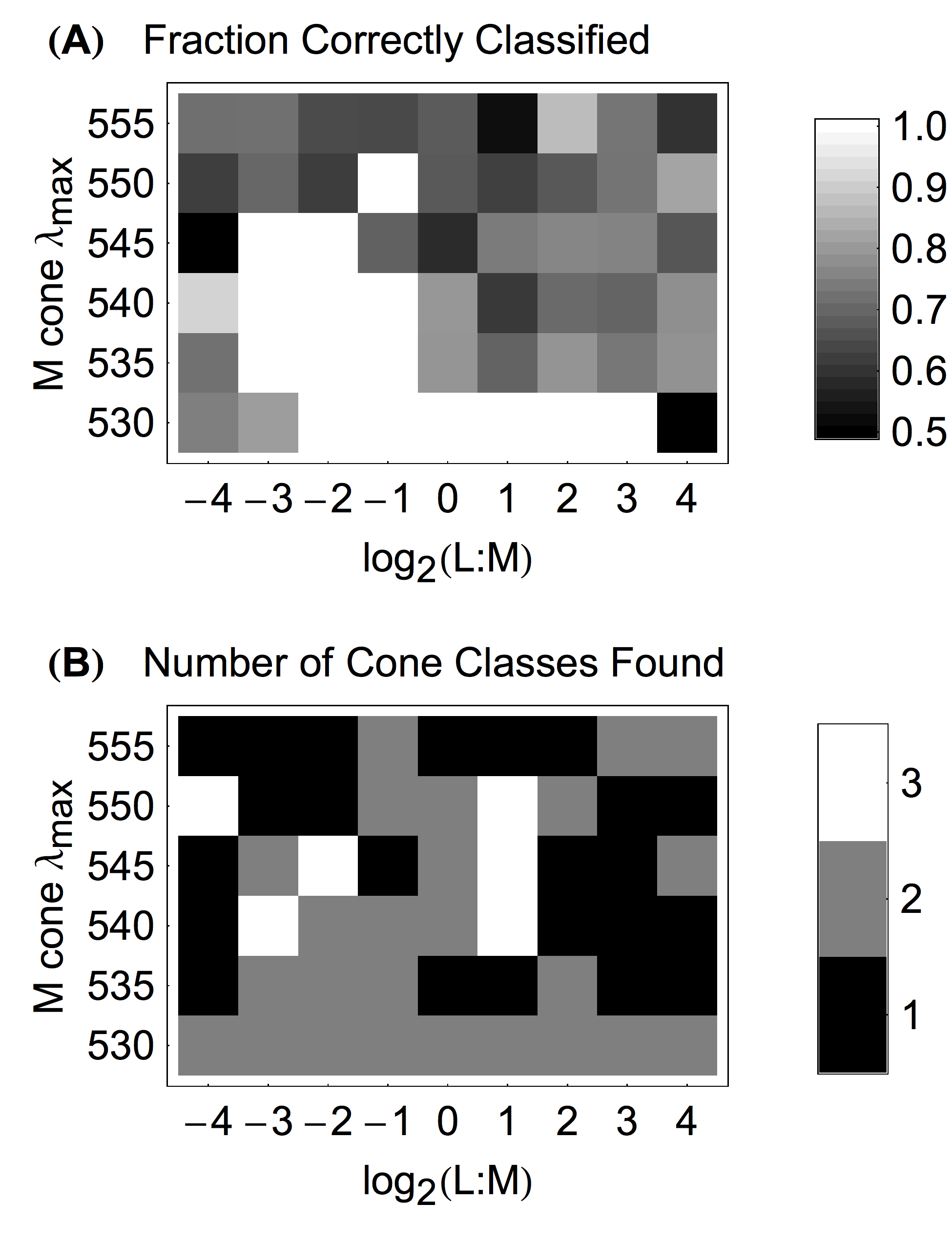

Supplement: Figure S8 — The algorithm performs more poorly when a cone-specific surround suppression is employed. Simulations were run with a Gaussian surround suppression ( cones, weight = 0.25) such that each cone was suppressed only by nearby cones of different type. L and M cones opposed each other, and S cone surrounds consisted of both L and M cones. In the case of retinas with extreme L∶M ratios, this often required that some cones have no surround (no nearby cones of a different type) while others had very strong surrounds (all nearby cones were of a different type). Each retinal mosaic was shown a different set of 2 million randomly-drawn natural image patches. (A) The fraction of cones correctly typed for a mosaic, for L∶M ratios ranging from 16∶1 to 1∶16 and for M cone values ranging from 530 nm and 555 nm. The number of cone classes was assumed to be 2. S cones were held at 6% of the cones and were given a value of 420.7 in all simulations. (B) The number of longer-wavelength-sensitive cone classes detected by the algorithm for each L∶M ratio and M cone . (TIFF) [file pcbi.1003652.s008.tiff]

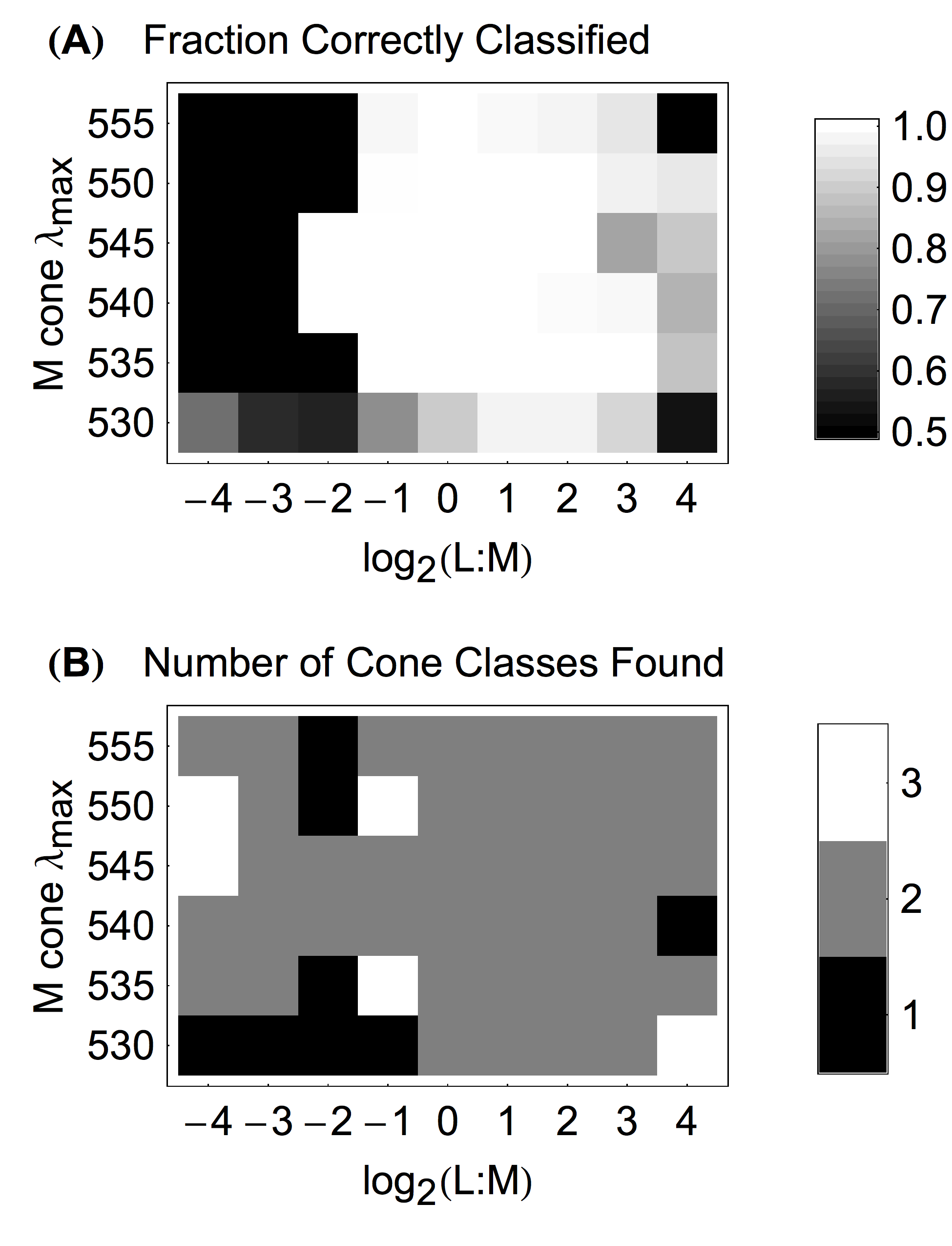

Supplement: Figure S9 — Information in the correlation matrix supports classification of L and M cones for tritanopes (no S cones). Results are shown for simulations of tritanopic mosaics with 4.5 million natural image patches. This is more image patches than were required for mosaics containing S cones. Although separation of L and M cones could be clearly observed in the 3D embeddings with 4.5 million image patches (see online supplement), the embeddings produced by the MDS algorithm were not aligned such that the first dimension of the MDS solution corresponded to the direction along which L and M cones separated. Nor, obviously, could we leverage the positions of the S cones to rotate the solution to produce such alignment as we did for simulations of mosaics with S cones. Accordingly, we manually rotated these embeddings before applying the flattening and classification steps of our algorithm so as to be able to quantify the separation between the L and M cones that could be achieved in a manner comparable with our other simulations. (A) The fraction of cones correctly typed for a mosaic, for various values of the L∶M ratio when the number of cone classes was assumed to be 2. The values for L and M cones were 558.9 and 530.3 nm, respectively. Note, however, the labeling of which cones are L and which are M by our algorithm becomes arbitrary in the absence of an S cone anchor. This arbitrariness was accounted for here as part of the manual rotation of the embedding. (B) The number of longer-wavelength-sensitive cone classes detected by the algorithm for each L∶M ratio. (TIFF) [file pcbi.1003652.s009.tiff]

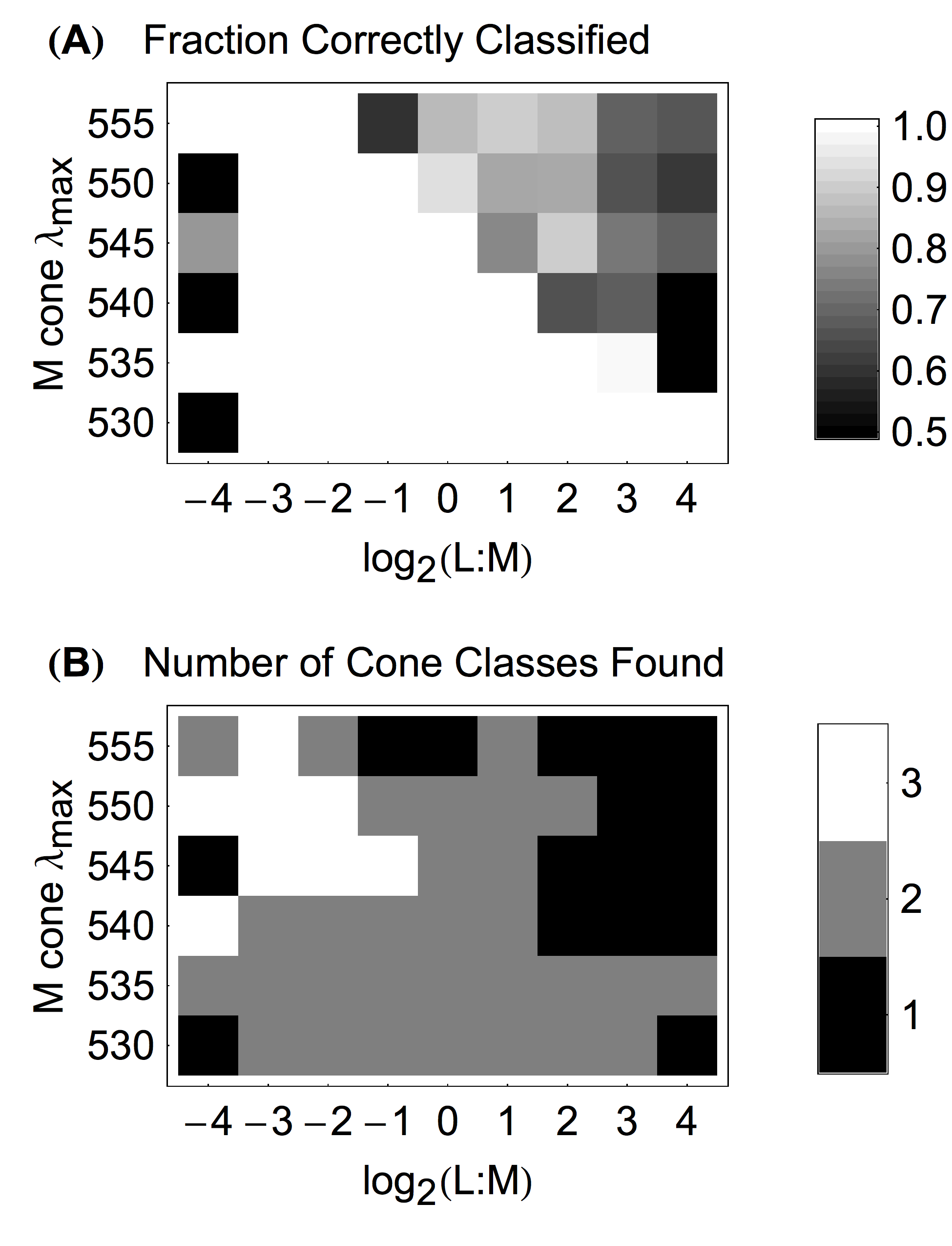

Supplement: Figure S10 — The algorithm performs well for images that exclude man-made objects. Performance is similar to those simulations in Figure 4, in which man-made items were not scrubbed from the image set, although there are a set of cases for M cone-rich retinas where three rather than two longer-wavelength-sensitive cone classes are detected. All retinal mosaics were shown a different set of 2 million randomly-drawn natural image patches. (A) The fraction of cones correctly typed for a mosaic, for various combinations of L∶M ratio and M cone value, when the number of longer-wavelength-sensitive cone classes was assumed to be 2. S cones were held at 6% of the cones and were given a value of 420.7 in all simulations. (B) The number of longer-wavelength-sensitive cone classes detected by the algorithm for each L∶M ratio and M cone value. (TIFF) [file pcbi.1003652.s010.tiff]

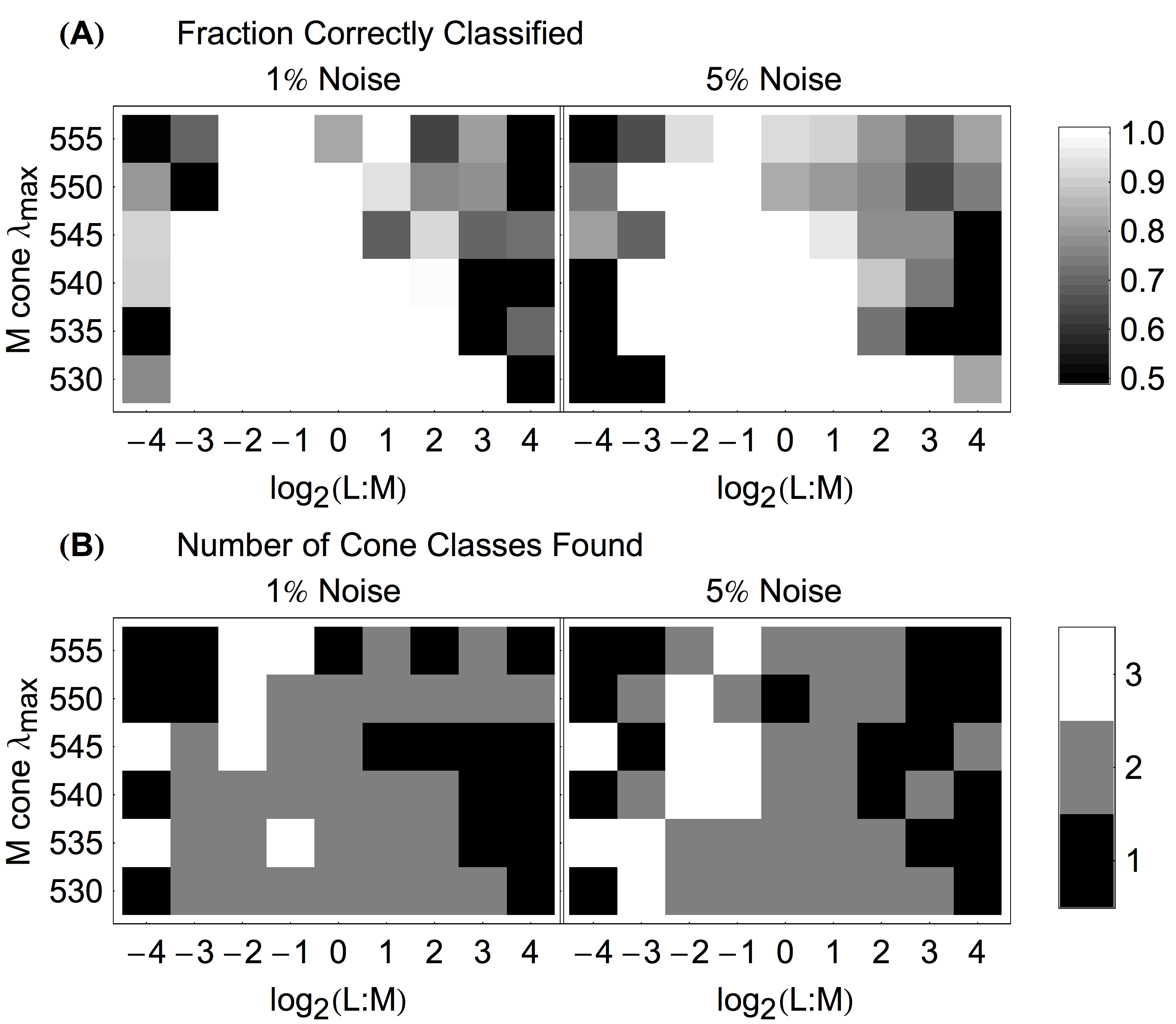

Supplement: Figure S11 — The algorithm is robust to noise. Noise was modeled by adding to each cone's response a random draw from a normal distribution whose standard deviation was 1% (left) or 5% (right) of the mean response from all cones. Each retinal mosaic was shown a different set of 2 million randomly-drawn natural image patches. (A) The fraction of cones correctly typed for a mosaic, for L∶M ratios ranging from 16∶1 to 1∶16 and for M cone values ranging from 530 nm and 555 nm. The number of cone classes was assumed to be 2. S cones were held at 6% of the cones and were given a value of 420.7 in all simulations. (B) The number of longer-wavelength-sensitive cone classes detected by the algorithm for each L∶M ratio and M cone . (TIFF) [file pcbi.1003652.s011.tiff]

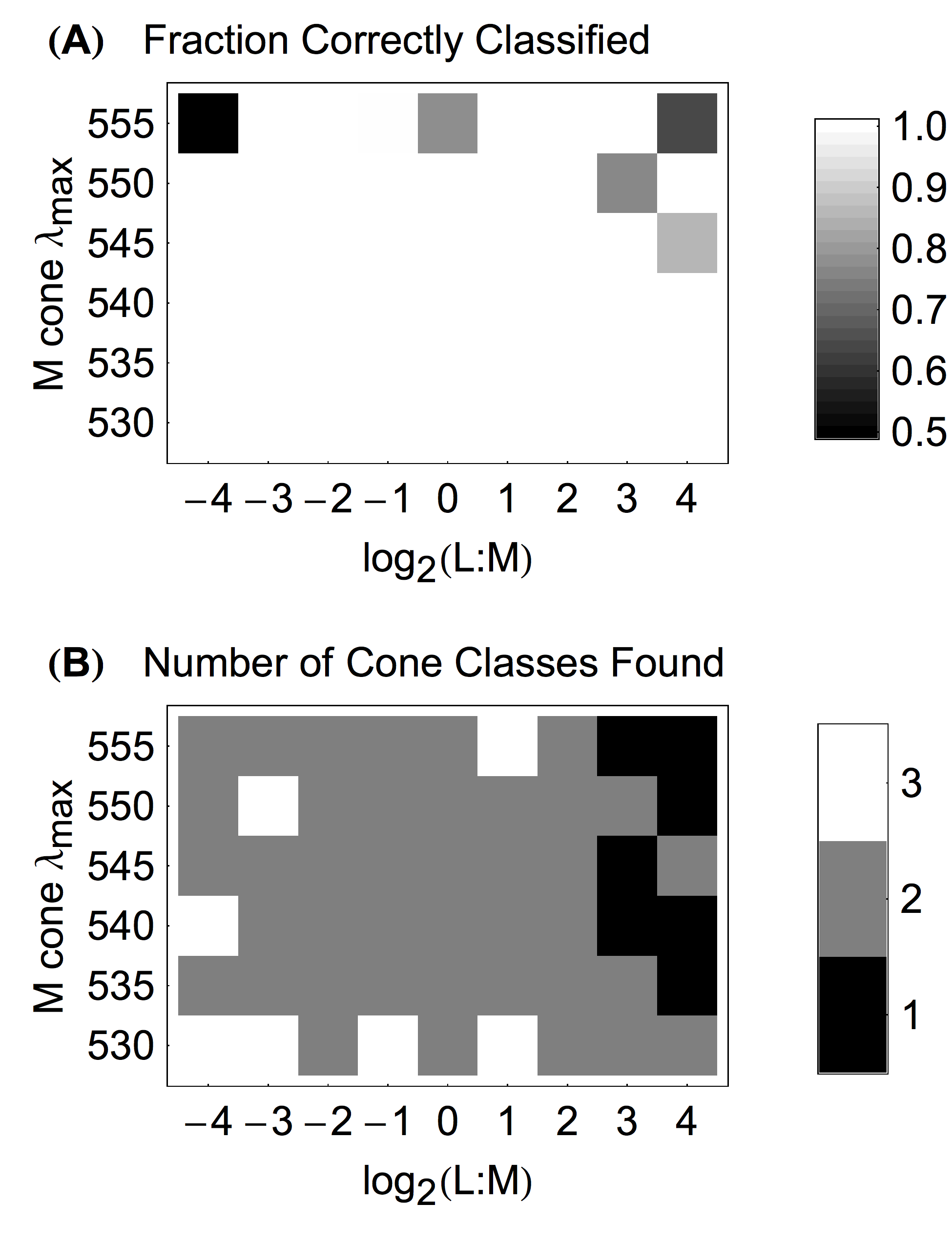

Supplement: Figure S12 — The algorithm performs well when images are blurred, although there are a few cases where three rather than two longer-wavelength-sensitive cone classes are detected. Blurring was accomplished by convolving each image with a 2D Gaussian with a standard deviation of 4 pixels. Each retinal mosaic was shown a different draw of 2 million natural image patches. (A) The fraction of cones correctly typed for a mosaic, for various combinations of L∶M ratio and M cone value, when the number of longer-wavelength-sensitive cone classes was assumed to be 2. S cones were held at 6% of the cones and were given a value of 420.7 in all simulations. (B) The number of longer-wavelength-sensitive cone classes detected by the algorithm for each L∶M ratio and M cone value. (TIFF) [file pcbi.1003652.s012.tiff]
